# POx-Lipids as an Alternative to PEG-Lipids? Multimethod Assessment of Chemistry and Structure

**DOI:** 10.1021/acs.analchem.5c07351

**Published:** 2026-03-13

**Authors:** Ekaterina Tsarenko, Ilya Anufriev, Caroline T. Holick, Tobias Klein, Stephanie Schubert, Nicole Fritz, Stephanie Hoeppener, Ulrich S. Schubert, Ivo Nischang

**Affiliations:** a Laboratory of Organic and Macromolecular Chemistry (IOMC), 9378 Friedrich Schiller University Jena, Humboldtstraße 10, 07743 Jena, Germany; b Jena Center for Soft Matter (JCSM), 9378 Friedrich Schiller University Jena, Philosophenweg 7, 07743 Jena, Germany; c Helmholtz Institute for Polymers in Energy Applications Jena (HIPOLE Jena), Lessingstraße 12-14, 07743 Jena, Germany; d Helmholtz-Zentrum Berlin für Materialien und Energie GmbH (HZB), Hahn-Meitner-Platz 1, 14109 Berlin, Germany

## Abstract

The characterization of polymer–lipid conjugates is essential for their successful application in pharmaceutical formulations, particularly in lipid nanoparticles (LNPs). While poly­(ethylene glycol) (PEG) lipids are widely used in LNPs, growing concerns over PEG immunogenicity have prompted the search for alternatives. Here, we investigate a series of poly­(2-ethyl-2-oxazoline) (PEtOx) lipids as promising PEG substitutes featuring biocompatibility, tunable hydrophilicity, and stealth-like properties. We apply a comprehensive, multimethod approach utilizing liquid chromatography (LC) and matrix-assisted laser desorption/ionization time-of-flight mass spectrometry (MALDI-TOF MS) to assess end group homogeneity, sample purity, and information about overall hydrophobicity/hydrophilicity of PEtOx-lipids compared to benchmark PEG-lipids used in COVID vaccines. We also demonstrate that under conditions of high sample purity only, hydrodynamic techniques such as analytical ultracentrifugation (AUC) become robust tools for analyzing conformational properties, aggregation behavior, and hydration of the polymers in aqueous environments. Though PEtOx-lipid analogues form micelles with lower aggregation numbers than PEG-lipids, they feature the same hydrodynamic size and values of hydration. The large levels of hydration of PEtOx-lipid micelles cannot be explained by hydrogen bonded water. Water present in the micelles constitutes a major part of their overall hydrodynamic volume and is located between the extended chain conformation of assembled polymers. Our study, with a unique multimethod approach, shows the importance of sample purity in high-end hydrodynamic analysis and paves the way for a quantitative replacement of PEG-based lipid conjugates by PEtOx-based lipid conjugates through a distinct set of properties to be considered for a particular application.

## Graphical abstract

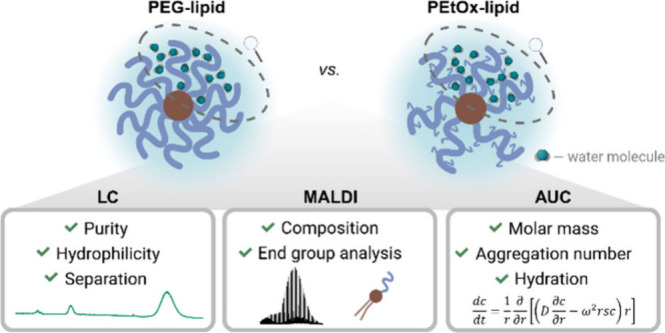

Poly­(ethylene glycol) (PEG) is a water-soluble polymer approved by the U.S. Food and Drug Administration (FDA) and the European Medicines Agency (EMA) for the use in the pharmaceutical industry and for drug delivery applications.^1,2^ Chemical modification of active pharmaceutical ingredients (APIs) or proteins with PEG (also known as PEGylation) showed improvement of pharmaceutical assets such as drug solubility, circulation time in the bloodstream, and prolongation of the half-life for the therapeutics to decelerate their clearance through metabolic pathways.^3^

The excessive use of PEG in biomedical applications by now resulted in reported immune responses against PEGylated therapeutics, with anti-PEG antibodies detectable in a large share of the human population (above 70%).^4,5^ Moreover, such antibodies were found also among patients who have never been treated with PEGylated medication, but have consumed or used products containing PEG.^6^ This leads to the loss of drug efficacy, accelerated blood clearance and, more seldomly, in appearance of severe allergic reactions.^6,7^

The emerging problems associated with PEG have boosted the search for alternative polymers for use in therapeutic applications.^8^ Among others, poly­(2-alkyl-2-oxazoline)­s (POx) are of particular interest. The tailorable hydrophobicity/hydrophilicity by the alkyl substituent in POxs is particularly appealing.^9^ For example, poly­(2-ethyl-2-oxazoline) (PEtOx) shows similar “stealth” behavior as PEGylated systems,^10^ namely shielding of APIs and drug delivery systems (DDS) from immune recognition and rapid clearance from the organs. At the same time, the extensive characterization of standard homologous series of polymers of PEtOx and PEG in solution by hydrodynamic techniques such as analytical ultracentrifugation (AUC), viscometry, and densimetry as well as light scattering methods already showed overall similar conformational properties in solution. However, at the same molar mass, PEtOxs feature smaller intrinsic viscosities (representing the hydrodynamic volume), larger diffusion coefficients, and slightly larger sedimentation coefficients than PEG. This all hints at their more compact nature in solution. It was claimed that careful adjustment of molar masses allows tailoring of the aforementioned properties such that PEtOx is a suitable candidate for PEG replacement in biomedical applications.^11^

Lipid nanoparticles (LNPs) are perhaps the most recent success of PEG and have gained more attention in manifold applications that, among others, include use as a promising DDS suitable for APIs, chemotherapy agents, and nucleic acid therapeutics.^12–14^ For example, modern vaccination strategies against COVID-19 used LNPs with PEG-lipids as carriers for enabling the delivery of messenger ribonucleic acid (mRNA) with a high efficacy. The successful delivery led to an immune response including the production of desired antibodies against the SARS-CoV-2 virus.^15–17^ The current FDA-approved LNP formulations contain several types of lipids, including ionizable, cationic, and PEGylated lipids (PEG-lipids).^18^ PEG-lipids in LNPs affect particle size and ζ-potential, have an impact on LNPs’ colloidal stability, and prevent undesirable interactions with serum proteins. Depending on the specific application, this reduces particle clearance from blood, resulting in their longer systemic circulation.^19,20^

However, the aforementioned elevated levels of vaccine-induced anti-PEG antibodies have been reported among patients.^21,22^ In search of suitable PEG alternatives for LNPs, several LNP systems, containing POx-lipids instead of conventional PEG-lipids, have been reported.^23–25^ For example, Kataoka and co-workers reported a novel approach for synthesis of heterotelechelic POx that were utilized in LNP formulations. Notably, the described LNPs containing POx-lipids revealed extended circulation after an intravenous injection in mice, confirming stealth behavior of POx-containing nanoassemblies. Also, the materials showed gene expression in the liver of mice comparable to that achieved with PEG.^23^ Recent studies underline broad efforts to tailor POx-based systems conjugated to lipids as advanced alternatives to PEG-lipids.^23–29^

Holick et al. developed a synthesis route toward a series of PEtOx-lipids, where ditetradecylamine was attached to the polymer chain via a succinate linker in a series of postpolymerization reactions.^25^ The prepared PEtOx-lipid series demonstrated cytocompatibility and increased transfection efficiency compared to LNPs formulated with PEG-lipids.^25^ Despite the synthesis and initial biological investigation of LNP systems utilizing POx as a PEG replacement, no detailed quantitative analysis of the purity and molecular and hydrodynamic characteristics of POx-lipids has been performed. However, these properties are essential in terms of quality assurance for the successful translation of novel materials to the pharmaceutical industry. Specifically and apart from the above-mentioned and broad efforts to find alternatives to PEG, much less attention has been focused on the in-depth analysis of alternatives with the opportunities of modern analytical chemistry at hand. We consider this important also, because studies related to the use of PEG alternatives would clearly benefit from quantitative information on physicochemical quality attributes of the polymers. This is essential for quality control and usage in later clinical trials. We open the door to this by techniques and their combination not commonly applied or established yet in the field. Perspectively, like PEG, POx should then enable stability of cargo in LNPs, allow for a reduced recognition from the immune system, and assist in delivery applications by their individual structure–property relationships manifested in the properties of the LNPs.

The desired quality attributes concern absolute values of the molar mass, end group identity, purity of the sample, hydrophobicity/hydrophilicity of the lipid conjugates, polymer solvent interactions, as well as solution structural properties of unimers and their aggregates in aqueous solution. The study of those properties requires the development of a multiplicity of analytical techniques, allowing for several orthogonal levels of insight, both chemically and physically.

While we do not directly study LNP behavior, our focus is centered on the POx-lipids whose physicochemical quality attributes are yet to be established to allow for an informed use in alternative drug delivery strategies including LNPs. We here report a quantitative study of the molecular properties of commercial PEG-lipids and a series of PEtOx-lipids (Scheme 1) by a combination of developed protocols including (i) a quantitative liquid chromatographic purity analysis with selectivity according to the end group identity, also resolving possible contaminants in the sample, and indicating overall hydrophilicity of the materials, (ii) a study of molecular hydrodynamic properties addressing the structure of isolated polymer–lipids in ethanol solution and their tendency to aggregate in the selective solvent water, and finally, for readily clean samples (iii) the quantitative estimation of the aggregation number and level of hydration from translational and rotational friction properties. For POx-lipids, we covered a range of degrees of polymerization below that of PEG-lipids and significantly above, to ensure usefulness of our analytical protocols for a range of molar masses that have already been reported in LNP research or become interesting in future research endeavors.^25^ We show that differences, such as polymer purity, its hydrophilicity/hydrophobicity, degree of aggregation, and hydration are quantitatively resolvable by the tools developed here. Such detailed characterization is essential not only for selecting suitable materials but also for ensuring the high levels of purity and batch-to-batch consistency required for future pharmaceutical development and good manufacturing practice (GMP) compliant production, addressing a suitable PEtOx-lipid replacement candidate to PEG.

**Scheme 1:**
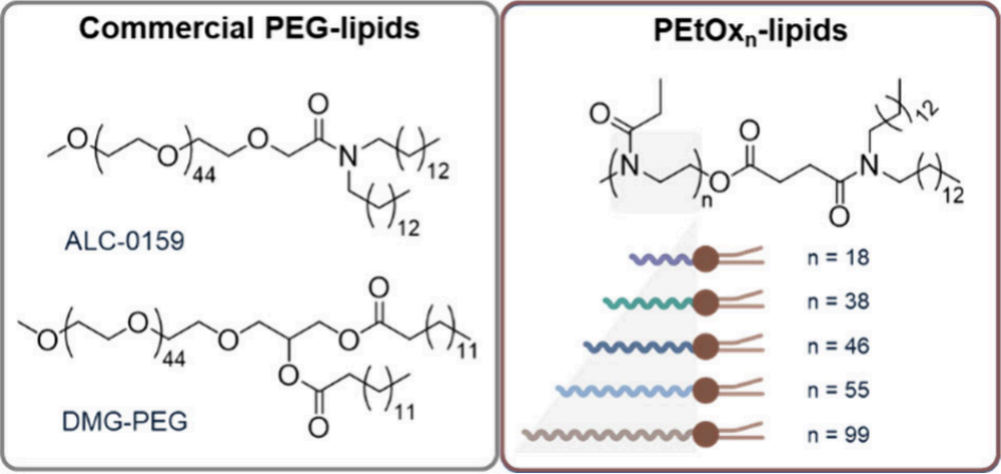
Representation of Benchmark Commercial PEG-Lipids as Well as a Series of Analogues, i.e., Poly­(2-ethyl-2-oxazoline)-Lipids (PEtOx_*n*_-Lipids), with the Degree of Polymerization (DP) of EtOx Monomers Indicated by *n*

## Experimental Section

### Materials

The commercial PEG-lipids ALC-0159 (α-[2-(ditetradecylamino)-2-oxoethyl]-ω-methoxy-poly­(oxy-1,2-ethanediyl)) and DMG-PEG (1,2-dimyristoyl-rac-glycero-3-methoxypolyethylene glycol-2000) were purchased from Biomol (Hamburg, Germany) and Merck KGaA (Darmstadt, Germany), respectively (Scheme 1). The synthesis of the PEtOx_*n*_-lipid series was previously described.^25^ Syntheses not published are described in the Supporting Information (section 1). In consequence of their detailed study, the synthesis was also optimized here for a selected PEtOx_*n*_-lipid chosen as a PEG mimic (vide infra). Ditetradecylamine was purchased from Ambeed (Arlington Heights, USA) as well as a high purity alternative received as a kind gift from Evonik (Hanau, Germany). LC-MS grade acetonitrile, methanol, and water as well as formic acid (FA, ≥99.9%) were purchased from VWR (Darmstadt, Germany). For hydrodynamic experiments, ethanol was obtained from Merck KGaA (Darmstadt, Germany). Ultrapure water was obtained from a Thermo Scientific Barnstead GenPure xCAD Plus water purification system (Thermo Electron LED GmbH, Langenselbold, Germany).

### Liquid Chromatography (LC)

For LC, an UltiMate 3000 Rapid Separation (RS) UHPLC chromatographic system from Thermo Fisher Scientific (Waltham, MA, USA) equipped with a diode array detector (DAD) and a universal Corona Veo charged aerosol detector (CAD) connected in series was utilized. For the CAD, the nebulizer temperature was set to 45 °C and data were collected at a 5 Hz acquisition frequency. The system was controlled by a Chromeleon 7.2 SR5 software, which was also used for data processing. The autosampler temperature was set to 19 °C and the column oven temperature was set to 35 °C. The injection volume was 5 μL and a flow rate of 1 mL min^–1^ was utilized. All samples were dissolved at a concentration of 1 mg mL^–1^ in methanol and were filtered over a 0.45 μm pore size polytetrafluoroethylene (PTFE) filter from AppliChrom (Oranienburg, Germany).

The details on the LC method establishment and optimization, including the column and eluent choice for polymer–lipid analysis, can be found in Supporting Information (section 2.1). The finally utilized gradient elution method on all samples comprised a Chromolith Performance RP-8 end-capped column (100 mm × 4.6 mm) from Merck KGaA (Darmstadt, Germany) as a stationary phase and acetonitrile/0.1% aqueous FA solution as mobile phase. The eluent composition was kept constant for 1 min at 20/80 (%, v/v) acetonitrile/0.1% FA, followed by an increase to 50% in 3.5 min and then ramped up to 98% within 15.5 min. After these 20 min, the acetonitrile content was kept constant at 98% for 10 min, after which the initial conditions of 20/80 (%, v/v) acetonitrile/0.1% FA were re-established within 2 min. Here, the column was re-equilibrated for 6 min prior to the next injection.

For fractionation, the CAD was disconnected from the chromatography system, and an automated Fraction Collector FT from Thermo Fisher Scientific (Waltham, MA, USA) was connected to the HPLC system directly after the DAD using a tubing with 191 μL delay volume.

### Matrix-Assisted Laser Desorption Ionization Time-of-Flight Mass Spectrometry (MALDI-TOF MS)

Matrix-assisted laser desorption ionization time-of-flight mass spectrometry (MALDI-TOF MS) measurements were carried out utilizing a rapifleX MALDI TOF/TOF instrument (Bruker Daltonik, Bremen, Germany). The instrument was equipped with a scoutMTP II ion source and a smartbeam 3D laser (λ = 355 nm). All spectra were measured in positive reflector mode. *trans*-2-[3-(4-*tert*-Butylphenyl)-2-methyl-2-propenylidene] malononitrile (DCTB; Sigma-Aldrich, Darmstadt, Germany) was used as a matrix supplemented with sodium trifluoroacetate (NaTFA; Sigma-Aldrich, Darmstadt, Germany) or sodium iodide (NaI; Sigma-Aldrich, Darmstadt, Germany). Spectra were recorded using the manufacturer’s software flexControl 4.0. Spectra evaluation and processing were performed using the flexAnalysis 4.0 software. Baseline subtraction and external calibration was performed using a poly­(methyl methacrylate) (PMMA) standard (Polymer Standards Service, Mainz, Germany) and a fleXstandard (Bruker Daltonik, Bremen, Germany). Details on the standard preparation as well as spotting can be found in the Supporting Information (section 1.2).

For MALDI-TOF MS measurements of LC fractions, the initial solvent was evaporated under reduced pressure. 20 μL of DCTB in THF was added to each fraction in the vial. Afterward, 1 μL of the matrix and salt mixture (20 μL DCTB and 5 μL NaTFA) was spotted on a MALDI sample target plate and allowed to air-dry. The sample was spotted five times on top of the matrix and salt mixture spot. After each spotting, the sample was allowed to air-dry. Afterward, the spots were covered once again with 1 μL of matrix and salt mixture and air-dried, and the target plate was introduced to the instrument.

### Electrospray Ionization Mass Spectrometry (ESI MS)

Electrospray ionization (ESI) mass spectrometry (MS) was measured on a micrOTOF Q-II mass spectrometer (Bruker Daltonik, Bremen, Germany). The instrument was equipped with an automatic syringe pump from KD Scientific for the sample infusion. The samples were dissolved in 10/90 (%, v/v) 0.1% aqueous FA/methanol. The ESI-Q-ToF mass spectrometer was operated in the positive ion mode at 4.5 kV with a desolvation temperature of 180 °C. Nitrogen was used as a nebulizer and drying gas. All fractions were infused using a constant flow rate (3 μLmin ^–1^) of sample solution. The instrument was calibrated in the *m*/*z* range of 50 to 3000 using a calibration standard (ESI-L Low Concentration Tuning Mix) received from Agilent Technologies (Waldbronn, Germany). All data were processed via the Bruker Data Analysis software version 4.2 (Bruker Daltonics, MA, USA).

### Viscometry

Viscometric studies were performed in ethanol and water as solvents with a Microviscometer (AMVn, Anton Paar, Graz, Austria) utilizing a capillary/ball combination.^11,30^ The ball times in solvent, *t*_0_, and in the polymer solution at certain concentration, *t*_*c*_, were measured in a glass capillary with an inner diameter of *d* = 0.9mm. The relative viscosity η_*r*_ = *t*_*c*_/*t*_0_ for all measurements was in the range 1.2 ≤ η_*r*_ ≤ 2.5. Measurements were conducted at a temperature of *T* = 20 °C and a capillary inclination angle of 50°. More details describing determination of the intrinsic viscosities, [η], of the samples from such measurements can be found in the Supporting Information (section 2.7.1).

### Densimetry

Density increment measurements were performed using a DMA 4100M densimeter (Anton Paar, Graz, Austria) in ethanol and water as solvents at a temperature of *T* = 20 °C. Partial specific volumes of the samples, υ, were determined through a procedure described elsewhere.^11,30,31^

### Analytical Ultracentrifugation (AUC)

Sedimentation velocity AUC (SV-AUC) experiments were performed with a ProteomeLab XL- I analytical ultracentrifuge (Beckman Coulter, Brea, CA) similar to as described previously.^11,30,32^ The AUC cells were assembled with double-sector Epon (for solvent water) or aluminum (for solvent ethanol) centerpieces. Those centerpieces led to a 12mm optical solution path length. The sectors were filled with ca. 440 μL of pure solvent (water or ethanol) in the reference sector and ca. 420 μL of the polymer–lipid solutions in the sample sector. After placing the cells in an eight-hole rotor (An-50Ti) and temperature equilibration at *T* = 20 °C, the experiments were conducted at a rotor speed of 42,000rpm. The radial- and time-resolved sedimentation velocity profile scans were recorded by the Rayleigh-interference optical detection system (RI) at a time interval of 3 min. The numerical sedimentation-diffusion analysis of the experimental sedimentation velocity data was performed in Sedfit (version 18.1) using the *c*(*s*) model.^33^ For AUC data representation, GUSSI software (version 2.1.6) was utilized in instances.^34^ Further details about the SV-AUC analysis procedures can be found in the Supporting Information (section 2.7.1).

### Dynamic Light Scattering (DLS)

Dynamic light scattering (DLS) in water was performed with a Zetasizer Nano-ZS (Malvern Instruments Ltd., Worcestershire, U.K.), equipped with a 663 nm He–Ne laser, at a temperature of *T* = 20 °*C*. Intensity fluctuations were recorded at a backscattering angle of 175 °. The *d*_*h*,*DLS*_ and the polydispersity index (PDI) were obtained by cumulant analysis of the corresponding decay functions and extracted diffusion coefficients.

### Cryogenic Transmission Electron Microscopy (cryo-TEM)

Cryo-TEM investigations were performed on a Titan Krios G4 (Thermo Fisher Scientific, Eindhoven, The Netherlands) system at an acceleration voltage of 300 kV. Samples were blotted onto a Quantifoil grid (R 2/2, Quantifoil, 8.5 μL of solutions with a concentration of ≈10mgmL^–1^) utilizing a Vitrobot Mark IV preparation unit (blotting time 1 s, offset −6 mm). Samples were vitrified utilizing liquid ethane as the cryogen. After blotting, the sample temperature was always maintained below −165 °C. The grids were transferred to autogrids and the autoloader system (Krios). Images were acquired with the Ceta camera of the Krios. Contrast adjustments and image analysis was performed by ImageJ (version 1.52a, National Institutes of Health, Bethesda, MD, U.S.). For the micelle size determination, several areas from different images containing >100 micelles showing a hexagonal arrangement, were processed by Fast Fourier Transform (FFT). The resulting pattern was utilized to estimate micelle size as described previously.^30^ Aiming at the analysis of hexagonally packed areas ensures that the micellar shell is included in the size determination. Visualization of the shells imposes natural difficulties, as their electron contrast remains generally low.

## Results and Discussion

### Polymer Entries

The PEtOx_*n*_-lipids were synthesized in a series of end group modifications at the ω-terminus (Scheme 1 and Supporting Information, section 1, Scheme S1, Figures S1–S5, Tables S1 and S2). The structure closely resembles that of ALC-0159, although the lipophilic moiety is attached through a succinate linker for PEtOx_*n*_-lipids analyzed in this report. Throughout this study, “*n*” denotes the degree of polymerization (DP) of EtOx monomers to PEtOx_*n*_. The DP indicated by “*n*” in the present manuscript was determined by proton nuclear magnetic resonance (^1^H NMR) spectroscopy after polymerization. The decisive step within the synthesis route toward the PEtOx_*n*_-lipids comprises the amidation of PEtOx_*n*_ with carboxylic acid end groups using ditetradecylamine. The PEtOx_*n*_-lipids were investigated on par with commercially available benchmark PEG-lipids, i.e., ALC-0159 and DMG-PEG (Scheme 1) for which literature data on hydrodynamic characteristics are available.^30^

### Sample Purity Analysis by LC of PEtOx-Lipids and Benchmark PEG-Lipids

After development and optimization using PEtOx_18_-lipid (Figure S6, Figure S7), the tailored LC method comprised a less hydrophobic C8 column operated with a binary mobile phase solvent composition of aqueous acetonitrile. An acidified aqueous mobile phase containing 0.1% (v/v) formic acid (FA) was used. To improve resolution and increase selectivity among eluting species, a gradient with a segmented change in the gradient slope of aqueous acetonitrile was utilized (Figure S7). This gradient programming of the mobile phase enabled complete elution of all the species in the sample. Compared to the simple linear gradient, the overall elution time window increased significantly with the segmented gradient, while the apparent spacing among species increased (Figure S7). Under identical chromatographic conditions for elution, the series of PEtOx_*n*_-lipids with different DP values was analyzed, and the elution behavior was compared to benchmark PEG-lipids (Figure 1).

**Figure 1:**
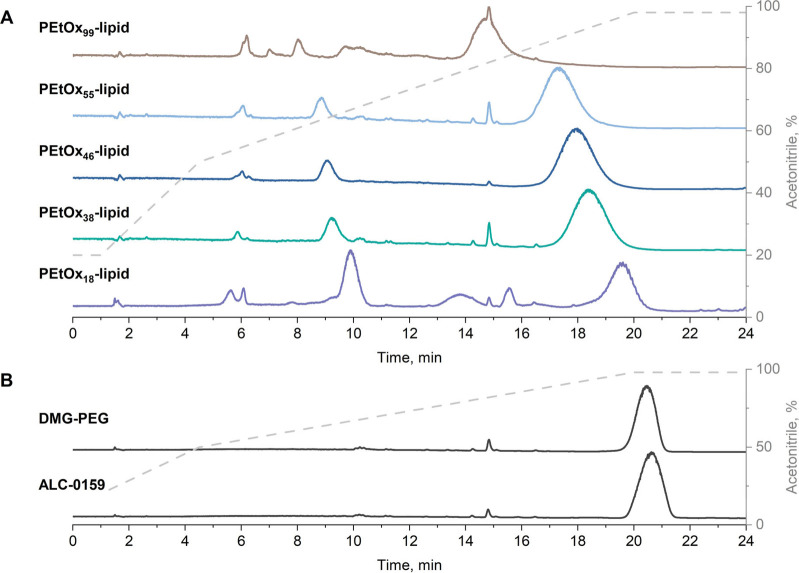
Normalized elution traces of (A) PEtOx_*n*_-lipid series and (B) commercial PEG-lipids (DMG-PEG and ALC-0159) recorded by CAD. Measurement conditions: mobile phase acetonitrile/0.1% FA (v/v), Chromolith Performance RP-8 end-capped column (100 mm × 4.6 mm), flow rate 1.0 mL min ^–1^, column oven temperature 35 °C, injection volume 5 μL. The gray dashed line indicates the acetonitrile gradient programming.

In contrast to PEG-lipids ALC-0159 and DMG-PEG, each PEtOx_*n*_-lipid eluted with several populations, evidencing the presence of different species within the sample (Figure 1A). To address the chemical composition distribution of the samples and the chemistry behind the multiplicity of signals in the elugram, MALDI-TOF MS was utilized. For that, fractions were collected during an LC run using an automated fraction collector. Figure 2 exemplifies the elugram of PEtOx_46_-lipid with indicated collected fractions (F1, F2, and F3) and the corresponding measured mass spectra. For all species, the difference between the two most abundant adjacent peak groups in the mass spectra equaled 99, which corresponds to one EtOx repeating unit which indicated that all species within one sample are PEtOx_46_ with different (un)­desired end groups (Figure 2B–D).

**Figure 2:**
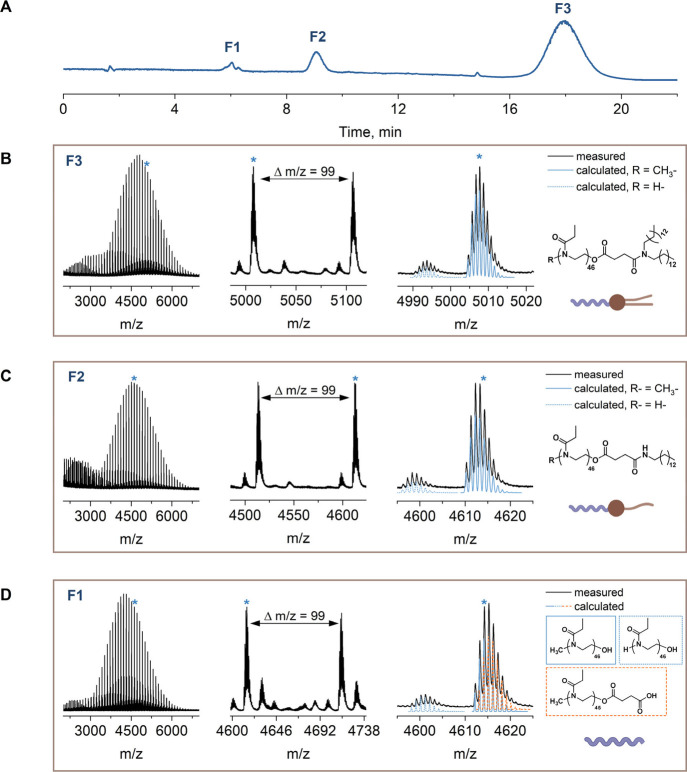
Composition analysis of PEtOx_46_-lipid by liquid chromatography, offline coupled to MALDI-TOF MS. (A) Separation of PEtOx_46_-lipid into the three main populations, F1, F2, and F3. (B–D) Full MALDI mass spectra of PEtOx_46_-lipid fractions with (B) F3, (C) F2, and (D) F1 with a zoom into the MALDI mass spectra of one EtOx repeating unit (Δ*m*/*z* = 99) each and overlay of the measured and calculated isotopic patterns for the indicated species. The measured spectra are plotted in black and the calculated isotopic patterns in color (blue and orange). Measurement conditions are specified in the Experimental Section.

Comparing the calculated isotopic patterns for PEtOx_46_ with the different ω end groups to the measured mass spectra allowed for composition analysis of the respective fractions and end group determination. In the case of PEtOx_46_-lipid, the most hydrophobic species (F3, *t*_*R*_ ≈ 18 min) refers to the final desired product, i.e., a PEtOx_46_ with a ditetradecylamine lipid end group (Figure 2B).

The species eluting earlier (F2, *t*_*R*_ of 8 to 11 min) match the isotopic pattern of PEtOx_46_ modified with tetradecylamine (Figure 2C). Apparently, the ditetradecylamine reagent contained the primary amine tetradecylamine as an impurity, as was verified by ESI-MS (Figure S8). In consequence, the presence of two types of amines in the commercial reagent led to the formation of significant amounts of PEtOx_46_ chains conjugated to tetradecylamine alongside the major desirable species where the ω end group was reacted with ditetradecylamine.

The most hydrophilic species (F1, eluting between 6 and 7 min) contained the PEtOx_46_ precursors void of the desirable lipid end group (Figure 2D). The calculated isotopic patterns for PEtOx_46_ with a hydroxy ω end group (PEtOx_46_-OH) and succinylated PEtOx_46_ with a carboxyl ω end group (PEtOx_46_-COOH) match the measured mass spectra. MALDI-TOF MS measurements for the other PEtOx_*n*_-lipids (Scheme 1) demonstrated analogous end groups for species eluting at the several identified elution time ranges (Figures S9–S12). Seen also is that mass spectrometric analysis of disperse polymers is reasonably possible up to 10.000 g mol^–1^ with the present method. For PEtOx_99_-lipid (Figure S12) it appears that accessibility of the fragmentation pattern in the data starts becoming more cumbersome in instances. We note that common to all mass spectra and fractions are reasonable amounts of H-initiated species. Those species are well-known in the preparation of POx.^35^

The commercial PEG-lipids eluted in one population at a higher acetonitrile content (Figure 1B). Their structure was assessed by MALDI-TOF MS, revealing high purity commercial samples with sufficient product homogeneity (Figures S13 and S14).

### Hydrophobicity of the PEG-Lipids and PEtOx-Lipids

With the method of analysis described, several interesting observations can be made. Regardless of the PEtOx-lipid DP, they eluted earlier than the PEG-lipids ALC-0159 and DMG-PEG (Figure 1A vs Figure 1B). Considering all species that were determined within a PEtOx_*n*_-lipid sample, an apparent hydrophobicity row was established by plotting the elution times of the indicated species from Figure 1 as a function of polymer DP, respectively (Figure 3).

**Figure 3:**
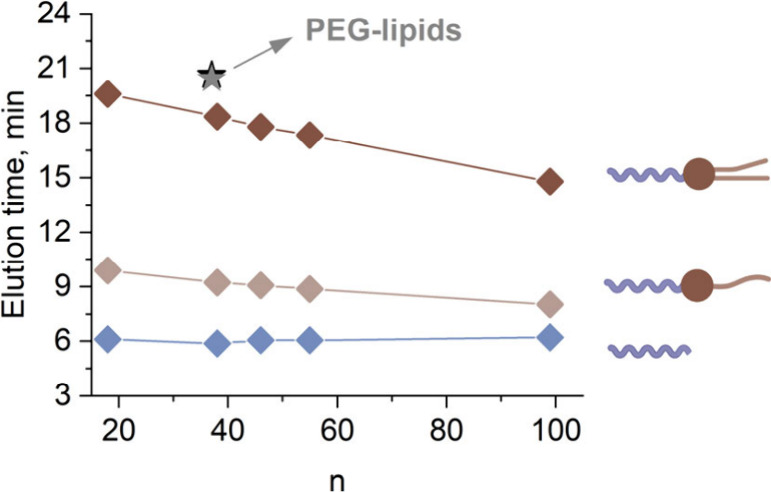
Trend of elution times against polymer degree of polymerization (DP) indicated by *n* for in-house made PEtOx_*n*_-lipids (ditetradecylamine) and commercial PEG-lipids (black star, ALC-0159; gray star, DMG-PEG). Shown also are the PEtOx_*n*_-lipids (tetradecylamine) as well as the precursors PEtOx_*n*_-OH and PEtOx_*n*_-COOH seen in the elugrams. Elution conditions are the same as in Figure 1.

The PEtOx_*n*_-lipids with end groups derived from ditetradecylamine eluted earlier as their DP value increased. This clearly evidences the diminishing impact of the hydrophobic lipid end group on polymer elution time. The species containing the less lipophilic tetradecylamine ω-end group showed much lower elution times with a similar, however attenuated, trend toward reduced elution times at increased chain length. The PEtOx_*n*_-OH and PEtOx_*n*_-COOH impurities eluting at around 6 min, feature identical DP values as the corresponding PEtOx_*n*_-lipids. Despite that, their elution times were independent of DP under the present chromatographic conditions, revealing that the elution of the PEtOx_*n*_-lipids is ruled by the lipid-modulated hydrophobicity of the individual polymers, instead of size exclusion. At increased DP value, the overall hydrophobicity of the conjugate decreased, leading to shorter elution times.

In either case, the protocol developed here allows for sufficient resolution to distinguish the different end groups and possible impurities in the sample. Based on the knowledge of chemical identity behind every collected fraction from MALDI-TOF mass spectra, a semiquantitative estimate of purity of the PEtOx_*n*_-lipids was determined as described in the Supporting Information (Table S3) together with brief statistical purity determination data for PEtOx_46_-lipid (Table S4, Figure S15). Particularly, as synthesized PEtOx_18_-lipid and PEtOx_99_-lipid showed apparent purity values of 43% and 63% only, while all others had purities above 80%.

Conclusively, the developed chromatographic protocol revealed that during synthesis of the respective PEtOx_*n*_-lipids, a manifold of resolvable species and impurities can be identified. Such information is difficult to get without proper chromatographic purity analysis and may help to optimize synthesis protocols. In the present case, the use of high purity ditetradecylamine void of tetradecylamine impurities seemed reasonable (vide infra). Furthermore, the hydrophilicity/hydrophobicity of the sample can be quantitatively judged on by a mere interpretation of elution times in reversed phase liquid chromatography under identical chromatographic conditions.

### PEtOx-Lipids vs PEG-Lipids: Quantitative Comparison

In view of the desired goal to replace PEG with suitable PEtOx candidates, a quantitative analysis of the structure–property relationships of the materials in solution is highly desirable. In polymer science, this requires absolute tools and methods such as high precision (molecular) densimetry, (molecular) viscometric measurements, and investigations enabled by an analytical ultracentrifuge allowing for assessment of sedimentation and diffusion coefficients. Initial hydrodynamic characterization of the materials including the above-mentioned techniques was performed in analogy to a procedure described elsewhere.^30^ Particularly, sedimentation-diffusion analysis by numerical solution of the Lamm equation for analyzing SV-AUC experiments was performed:

1 $$\frac{dc}{dt}=\frac{1}{r}\frac{\partial }{\partial r}\left[\left(D\frac{\partial c}{\partial r}-\omega^{2}rsc\right)r\right]$$

where *c* is the concentration, *t* is the time, *r* is the radius, *D* is the diffusion coefficient, ω is the angular velocity of the rotor, and *s* is the sedimentation coefficient. The radial and time-resolved concentration profiles were numerically solved via the *c*(*s*) model in Sedfit,^33^ making use of the hydrodynamic equivalent sphere concept.

The diffusion coefficients, *D*, under conditions of sedimentation are defined as

2 $$D=\frac{kT{\left(1-\upsilon \rho_{0}\right)}^{1/2}}{{\eta_{0}}^{3/2}9\pi \sqrt{2}{\left(f/f_{sph}\right)}^{3/2}{\left(s\upsilon \right)}^{1/2}}$$

where *k* is the Boltzmann constant, *T* is the temperature, υ is partial specific volume, η_0_ and ρ_0_ are solvent viscosity and density, respectively. The *c*(*s*) model in Sedfit returns a differential distribution of sedimentation coefficients and weight-average translational frictional ratios, *f*/*f*_*sph*_. In practice those parameters are investigated for a series of dilutions to obtain their estimate at infinite dilution, i.e., (*f*/*f*_*sph*_)_0_ or *D*_0_, and *s*_0_.^11,30,32,36^

Detailed information about the analysis including investigation of all PEtOx_*n*_-lipids can be found in the Supporting Information (section 2.7, Figures S18–S25, Tables S6 and S7). There are some general trends in the data such as increasing intrinsic viscosities, [η], increased sedimentation coefficients, *s*, increased translational frictional ratios, *f*/*f*_*sph*_, and a slightly decreasing partial specific volume, υ, at an increased polymer DP (Table S6). The latter stems from a vanishing impact of the lipophilic end group upon increased polymer DP. Also, polymers forming micelles in water (vide infra) appear to have increased [η], *s*, *f*/*f*_*sph*_ values, and a slightly decreasing partial specific volume, υ, at increased polymer DP (Table S6, Figure S20). Interestingly, an overall trend of increased hydration values in water at increased polymer DP, particularly if [η]-values are used for their calculation, is observed (Figure S25, Table S7).

Despite the general trends, we note discrepancy of the detailed hydrodynamic characteristics in the set of samples as opposed to previous studies of PEG-lipids,^30^ which are worthwhile discussing. Before doing so, some general considerations and scientific relations can be formulated. Our experiments provide access to the (intrinsic) sedimentation coefficients, [*s*], (intrinsic) diffusion coefficients, [*D*], as well as values of the intrinsic viscosity, [η], as prime hydrodynamic characteristic of the PEtOx_*n*_-lipids and their assemblies in solutions. While [η] is directly estimated by rotational friction experiments, [*s*] and [*D*] are derived from *D*_0_ and *s*_0_ of SV-AUC experiments and by utilizing the following equations:

3 $$\left[s\right]=\frac{s_{0}\eta_{0}}{\left(1-\upsilon \rho_{0}\right)}$$

4 $$\left[D\right]=\frac{D_{0}\eta_{0}}{T}$$

All characteristics, [η], [*s*], and [*D*], of the same sample relate to the hydrodynamic size, respectively, dimensions in solution. They can be united in the so-called hydrodynamic invariant *A*_0_:^11, 30, 32, 37, 38^

5 $$A_{0}={\left(R\left[s\right]{\left[D\right]}^{2}\left[\eta \right]\right)}^{1/3}$$

From its very fundamental origin, *A*_0_ is indicative of the polymer and colloid system at hand and typically does not vary in a homologous series of synthetic polymers.^37,38^ Also, *A*_0_ can be utilized to indicate the accuracy of the obtained hydrodynamic characteristics from the measurements of translational ([*s*] and [*D*]) and independently determined rotational friction, [η].

In the present study, sedimentation-diffusion analysis of SV-AUC experiments was performed. This leads one to values of sedimentation, *s* (consequently [*s*], eq 3), and translational frictional ratios, *f*/*f*_*sph*_ (respectively *D*, eq 2, or consequently [*D*], eq 4), based on numerical solution of the Lamm eq (eq 1).^33^ In the present analysis it is important to understand that both *s* and *D* (respectively *f*/*f*_*sph*_, eq 2) are determined from a SV-AUC experiment. The accuracy of those estimates needs to be benchmarked by an orthogonal hydrodynamic technique since we deal (i) with synthetic polymers being different species of what the software was developed for (particularly proteins and other biomacromolecules), (ii) those systems feature inherent dispersity in their molar mass, and (iii) the polymers show end group inhomogeneity as clearly shown by our LC data (Figure 1A). To benchmark *D* and *s* estimations from SV-AUC data with the apparently determined partial specific volumes, we use the rotational friction based intrinsic viscosity, [η], measured on the bulk sample with a capillary ball combination.^11,32,38^

Translational frictional ratios, *f*/*f*_*sph*_, are both affected by hydration, δ_*AUC*_, and shape anisotropy^39^ or the so-called Perrin friction factor, *P*, leading to the following fundamental relationship:^30,40–44^

6 $$f/f_{sph}=P{\left(\frac{\delta_{\mathrm{AUC}}}{\upsilon \rho_{0}}+1\right)}^{1/3}$$

For a spherical shape only, *P* = 1, hydration values from SV-AUC analysis, δ_AUC_, can be straightforwardly calculated:

7 $$\delta_{\mathrm{AUC}}=\left({\left(f/f_{sph}\right)}_{0}^{3}-1\right)\upsilon \rho_{0}$$

To not only rely on hydration values from SV-AUC data, the intrinsic viscosity, [η], can be used. For spherical anhydrous particles it is fundamentally defined by Einstein’s relation, [η] = 2.5υ.^45–47^ In case of hydrated spherically shaped macromolecules or particles, [η] is defined by^30,41,48^

8 $$\left[\eta \right]=2.5\upsilon +2.5\frac{\delta_{\mathrm{visco}}}{\rho_{0}}$$

Equation 8 implies that experimentally determined [η] values beyond that of an anhydrous sphere of the same partial specific volume as the colloid, 2.5υ, must consequently stem from hydration, δ_visco_, with the solvent of density ρ_0_.^30,41^ Rearranging eq 8 leads to hydration in case of spherically shaped colloids assessable by intrinsic viscosity measurements:

9 $$\delta_{\mathrm{visco}}=\left(\frac{\left[\eta \right]}{2.5}-\upsilon \right)\rho_{0}$$

Equations 7 and 9 result in dimensionless values of hydration of objects in solution that should be interpreted as the mass of water (or solvent) in g per g of material (*g*/*g*). In other words, nonhydrated spherical (isotropic) materials would have a frictional ratio of one and no hydration term (eq 7) and an intrinsic viscosity according to Einsteins relation, 2.5υ, and no hydration term (eq 9). Exceeding values lead to hydration terms, clearly discernible in the present work. With the above-mentioned relations (eqs 5–9), the results of our hydrodynamic investigation can be set in context and interpreted in a more fundamental fashion, enabling us to trace origin of yet unknown potential errors in hydrodynamic analysis discussed above.

Figure 4A shows an elugram of the PEtOx_46_-lipid that has an apparent monolipid impurity. This polymer was also analyzed by intrinsic viscosity, [η], measurements by the Huggins extrapolation plots in Figure 4C in ethanol (dark gray squares) and water (light gray triangles). We found the typical linear behavior in ethanol and nonlinear behavior in selective solvent water. This indicates structural changes of the polymer system when changing the solvent. Differential distributions of intrinsic sedimentation coefficients, [*s*] (eq 3), from sedimentation-diffusion analysis by the *c*(*s*) model in Sedfit showed relatively small [*s*]-values in ethanol (Figure 4D, dark gray trace) and a shift toward an order of magnitude larger values of [*s*] in water (Figure 4D, light gray trace), clearly hinting at the formation of aggregates (Table S8). Overall, sedimentation-diffusion analysis showed an acceptable level of residuals for these types of systems (Figure S26A). Investigations by cryo-TEM in water revealed assemblies in solution that also exhibited a clear structural uniformity indicative of well-defined spherical micellar structures (Figure 4E).

**Figure 4:**
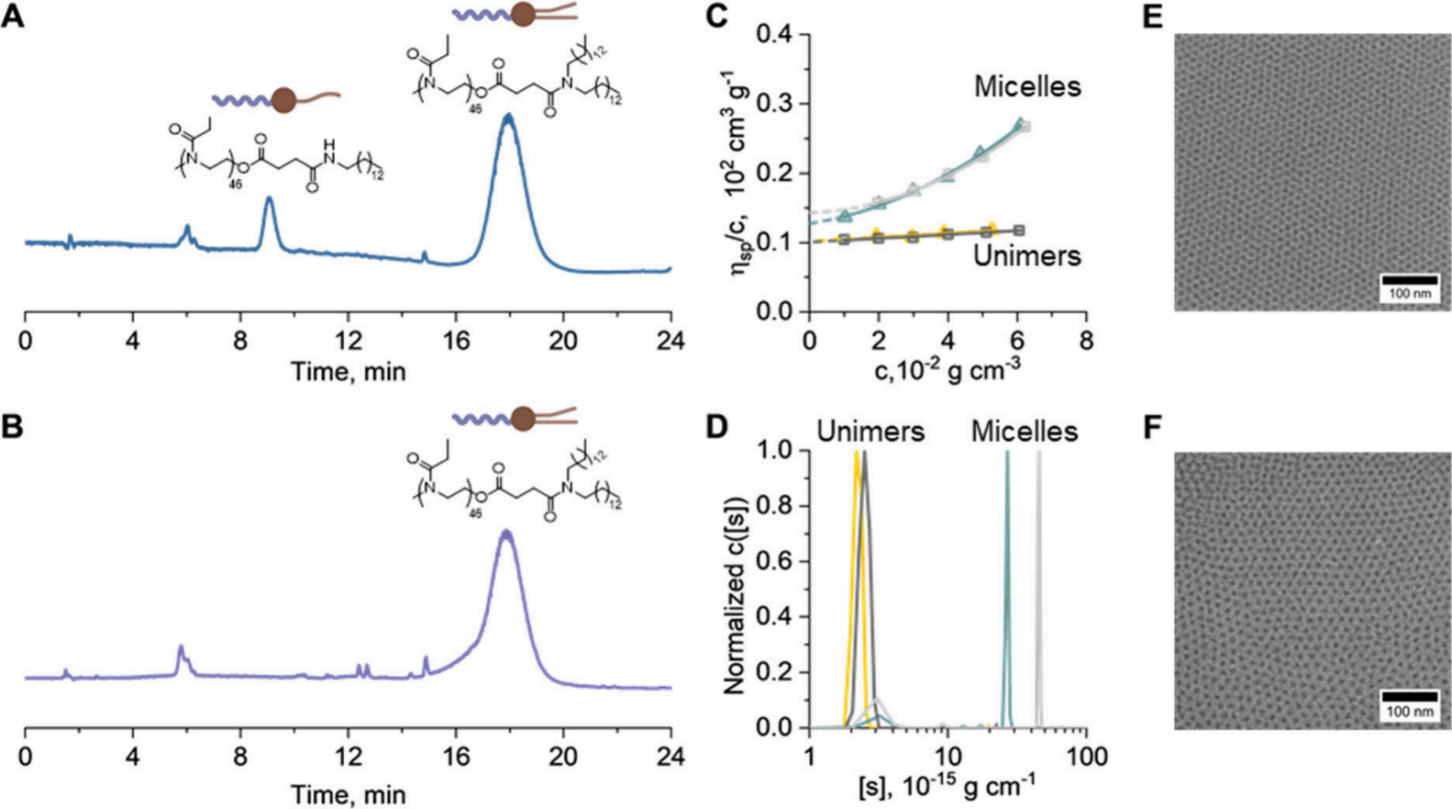
Results of experimental studies of PEtOx_46_-lipid and PEtOx_46_-lipid_clean_. Normalized chromatographic elution traces of (A) PEtOx_46_-lipid and (B) PEtOx_46_-lipid_clean_ recorded by CAD. (C) Huggins extrapolation plots of PEtOx_46_-lipid in ethanol (dark gray symbols and extrapolation) and water (light gray symbols and extrapolation) as well as PEtOx_46_-lipid_clean_ in ethanol (yellow symbols and extrapolation) and water (green symbols and extrapolation). (D) Normalized differential distributions of intrinsic sedimentation coefficients, *c*[s], of PEtOx_46_-lipid in ethanol (dark gray line) and in water (light gray line) as well as PEtOx_46_-lipid_clean_ in ethanol (yellow line) and in water (green line). Cryo-TEM images of (E) PEtOx_46_-lipid and (F) PEtOx_46_-lipid_clean_ in water.

In ethanol, *A*_0_ (eq 5) of PEtOx_46_-lipid assumes a value, on average being on the upper end of flexible chain macromolecules^37,38^ as well as comparable to PEG and the commercial PEG-lipids (Table S8). This indicates their similar conformational properties in solution that have been demonstrated before in extensive studies.^11,30,32,36^ Interestingly, however, values of *A*_0_ (eq 5) for the apparent aggregates in water indicated very similar values as in ethanol (Table S8). This is surprising because of the substantial structural change of the materials, forming micellar structures in water compared to unimolecularly dissolved polymer chains in ethanol (Figure 4C–E, Table S8).

A critical view on sedimentation-diffusion analysis in water should consider the translational frictional ratio, *f*/*f*_*sph*_, that is utilized in the definition of the diffusion coefficient, *D*, respectively derived under conditions of sedimentation through sedimentation-diffusion analysis (eqs 1 and 2). *D* is highly important in the hydrodynamic invariant, *A*_0_, due to its squared influence (eq 5). At the same time, *f*/*f*_*sph*_-values are deterministic for values of hydration of the materials through eq 6. The hexagonal arrangement seen for the PEtOx_46_-lipid in cryo-TEM is a consequence of the uniformity of the particle sizes, supporting the assumption of well-defined sizes (Figure 4E). However, unreasonable values of *A*_0_ for neither solid impermeable spheres^37,38^ nor solvent-permeable hydrated spheres are obtained as for the PEG-lipids (Table 1).^30^ At the same time utilizing *f*/*f*_*sph*_-values for calculating hydration (eq 7), resulted in a much smaller value (δ_AUC_ = 1.62g/g) than that derived from viscometric measurements (eq 9) of δ_visco_ = 4.59g/g (Table 1). Therefore, from sedimentation–diffusion analysis, micelles having an apparent molar mass corresponding to *M*_*s*,*f*_ = 377 000 g mol^–1^ (eq S6) are calculated. By dividing through the unimer molar mass determined in ethanol, an aggregation number of *N*_agg_ = 85 is estimated (Table 1).

**Table 1:** Hydrodynamic Invariants *A*_0_ (Eq 5), Values of Hydration δ_AUC_ (Eq 7) and δ_visco_ (Eq 9), Molar Masses *M*_*s*.*f*_ (Eq S6), and Aggregation Numbers *N*_agg_, of PEtOx_46_-Lipid, PEtOx_46_-Lipid_clean_, and Previously Studied Commercial PEG-Lipid Systems ALC-0159 and DMG-PEG, All Investigated in Water

sample	*A*_0_	δ_AUC_	δ_visco_	*M*_*s*. ***f***_	*N*_agg_
	10^–10^ g cm^2^ s^–2^ K^–1^ mol^–1/3^	g g^–1^	g g^–1^	g mol^–1^	
PEtOx_46_-lipid	3.78	1.62	4.59	377000	85
PEtOx_46_-lipid_clean_	2.77	4.94	4.13	254000	79
ALC-0159^a^	2.75	4.70	3.98	224000	112
DMG-PEG^a^	2.78	4.90	3.96	269000	128

a Values for ALC-0159 and DMG-PEG are published elsewhere.^30^

One may postulate that the above situation, based on the judgment of *A*_0_- and δ_AUC_-values, is likely associated with inadequate values of diffusion coefficients, respectively *f*/*f*_*sph*_-values, from sedimentation diffusion analysis (eq 2). An origin could be the impure sample that was identified by our LC measurements (Figure 1, Figure 2, and Figure 4A). While dispersity in polymers is also known to impact accurate molar mass estimations,^38,41^ our PEtOx_46_-lipid shows significant end group inhomogeneity as an inherent (molecular) chemical feature (Figure 4A). However, the multiplicity of species does not become apparent from viscometric measurements (Figure 4C), an AUC analysis (Figure 4D, Figure S26A), or cryo-TEM investigations in water (Figure 4E).

To check the above hypothesis and the developed tool of LC, we worked on a sample that is void of the major impurity found in our chromatographic investigations (PEtOx_46_-lipid with end groups based on tetradecylamine next to ditetradecylamine) and to which our polymer and colloid analysis techniques are somewhat insensitive, respectively return inconsistent values (vide supra). Therefore, next to the so far investigated and detailed PEtOx_46_-lipid, a new PEtOx_46_-lipid_clean_ was synthesized (Supporting Information, Section 1.3). This was enabled by utilizing high purity ditetradecylamine, quality-controlled by LC and MALDI-TOF MS (vide supra). The newly synthesized PEtOx_46_-lipid_clean_ had a similar elution time as the desirable fraction of the previous PEtOx_46_-lipid sample (Figure 4B vs Figure 4A). Because the primary amine in the reactant was absent, the tetradecylamine-lipid impurity was absent in the sample (Figure 4B, Figure S16). A purity value of 92.4 ± 0.5% was determined via LC (Table S5 and Figure S17).

As the next step, PEtOx_46_-lipid_clean_ was investigated using hydrodynamic methods. The Huggins plot changes from linear behavior to nonlinear behavior when changing the solvent from ethanol to water (Figure 4C). The concentration dependence of viscometric measurements of PEtOx_46_-lipid_clean_ does not change much in comparison with the PEtOx_46_-lipid. Also, we determine very similar values of intrinsic viscosities, [η], as the factually impure PEtOx_46_-lipid, evidencing that [η]-estimations on the bulk sample are an insensitive measure of sample purity. Sedimentation-diffusion analysis in ethanol resulted in similar estimates of translational frictional ratios, *f*/*f*_*sph*_, and intrinsic sedimentation coefficients, [*s*], of PEtOx_46_-lipid_clean_ as PEtOx_46_-lipid (Figure 4D, yellow trace vs dark gray trace). Apparently, AUC (alongside viscometric) analysis cannot distinguish PEtOx_46_-lipid and PEtOx_46_-lipid_clean_ sample in terms of the basic hydrodynamic characteristics in ethanol (Figure 4C,D, Table S8), leading to similar values of the hydrodynamic invariant (eq 5) from sedimentation-diffusion analysis (Table S8, Figure 4D). In water, intrinsic sedimentation coefficients of the major population of PEtOx_46_-lipid_clean_ decreased compared to PEtOx_46_-lipid (Figure 4D, green trace vs light gray trace). PEtOx_46_-lipid_clean_ featured increased translational frictional ratios *f*/*f*_*sph*_ compared to PEtOx_46_-lipid (Figure S28B, Table S8). The quality of analysis does not distinguish PEtOx_46_-lipid_clean_ from that of PEtOx_46_-lipid as demonstrated for a similar concentration (Figure S26B vs Figure S26A). Cryo-TEM images of the sample reveal a very similar hexagonal arrangement of micellar spherical structures of PEtOx_46_-lipid_clean_ (Figure 4F).

Interestingly, while we have a similar *A*_0_ for the PEtOx_46_-lipid and PEtOx_46_-lipid_clean_ in ethanol (barely resolvable differences in hydrodynamic behavior), the resulting value of *A*_0_ is 2.77 g cm^2^ s^–2^ K^–1^ mol^–1/3^ in water for PEtOx_46_-lipid_clean_ (Table 1). Such a value of *A*_0_ suggests existence of solvent-permeable hydrated spheres in solution. Also, such value is fully consistent with an independent study on commercial PEG-lipid systems in water (Table 1)^30^ that are void of impurities (Figure 1). At the same time, values of hydration for the micelle system of PEtOx_46_-lipid_clean_ based on frictional ratios (eq 7) are correlated with those by independent intrinsic viscosity measurements (eq 9). Those values are now also fully consistent with those of the benchmark PEG-lipids (Table 1).^30^ This shows that fully interrelated hydrodynamic characteristics are possible to extract from the sedimentation–diffusion analysis. However, it underscores SV-AUC as a self-sufficient tool in polymer and colloid analysis only at sufficient sample purity. As consequence, all colloid analysis techniques (SV-AUC, DLS, cryo-TEM, viscometry) yield very similar values of the effective size of PEtOx_46_-lipid_clean_ (Table S9), consistently identifying solvent permeable, hydrated spheres from assembled polymer material. A molar mass of *M*_*s*,*f*_ = 254 000 g mol^–1^ (eq S6) and an aggregation number of *N*_agg_ = 79 is determined (Table 1).

Based on the above quantitative and fully consistent results of the interrelation of hydrodynamic characteristics from studies on PEtOx_46_-lipid_clean_, we can draw further conclusions on the sample system and our initial motivation of designing a PEtOx-based lipid replacement to a benchmark PEG-lipid. The aggregation number, *N*_agg_, of the PEtOx_46_-lipid_clean_ was found to be *N*_agg_ = 79, which is lower than the average aggregation number for the commercial PEG-lipid systems (*N*_agg_ = 120), at a very similar hydrodynamic size (Figure S29, Table S9) and virtually identical values of hydration (Table 1).

The similar hydrodynamic size and values of hydration including molar mass (Tables 1 and S9) suggest very similar hydrodynamic characteristics of our PEtOx_46_-lipid_clean_ when compared to the benchmark PEG-lipids in water; i.e., hydrodynamically they are not distinguishable by their effective properties (Table 1). At the same time, such hydrodynamic equivalency is realized by a lower number of polymer chains, assembling into a PEtOx_46_-lipid_clean_ micelle system when compared to the benchmark PEG-lipids. In consequence, overall properties predestine the here studied PEtOx in studies concerning alternate drug delivery applications utilizing micellar systems and further exploration in LNP formulations void of PEG.

While the fundamental hydrodynamic properties of POx-lipids can be tailored to a PEG-lipid mimic on a molecular basis, the chemical structure of the POx backbone is different from that of the PEG, which also results in overall more hydrophilic properties of POx-lipids compared with PEG-lipids as judged from chromatographic elution (Figure 3). Therefore, the possible interactions of POx-lipids with other components in LNPs are yet to be investigated,^49^ also with regard to their correlation to biological performance where preclinical studies already exist.^24,25,29^ Such studies could greatly benefit when using POx-lipids with suitable and well-understood molecular characteristics compared to PEG, and foremost, that sufficient purity, matching that of PEG, is ensured. Guidelines to enable this are established here.

## Conclusions

In the present work, we applied a comprehensive set of orthogonal analytical techniques for characterization of novel polymer–lipid systems, researched as alternatives to the PEG-lipids in lipid nanoparticle formulations. Liquid chromatographic purity analysis proved essential to identify the end group homogeneity across the sample, revealing the influence of the degree of polymerization on elution time and setting in context the sample hydrophobicity of PEtOx-lipids when compared to benchmark PEG-lipids. Utilization of MALDI-TOF MS of elution fractions from high performance separation enabled identification of the different end groups in the material and allowed for comprehensive purity assessment.

We also demonstrated for the first time that, although AUC is considered a quantitative absolute tool in the hydrodynamic analysis of synthetic polymers and colloids, errors on simultaneous determination of sedimentation and diffusion coefficients of new research entries of synthetic materials should not be underestimated. Such errors can be tracked by a fundamental interrelation of hydrodynamic characteristics, pinpointing issues in sedimentation-diffusion analysis, not necessarily becoming apparent by the sole inspection of AUC data and modeling results. In our case, the origin of issues with sedimentation–diffusion analysis could be tracked to poor end group homogeneity identified by liquid chromatography, addressing the chemistry of materials. Quantitative hydrodynamic analysis of the polymers aiming at their physicochemical structure in an AUC setting is sensitive to errors. Though rarely performed in combination, we demonstrated that liquid chromatography is essential in the analysis of polymers concerning their composition and end group homogeneity that critically affect their chemical quality attributes as well as their derived physicochemical structure in solution.

Only if chemical composition homogeneity of polymers is met, substantially drawn quantitative conclusions on molecular hydrodynamic characteristics, conformational properties, and formation of aggregates in solution further underpin that PEtOx-lipid systems can be tailored to a suitable mimic for commercially available PEG-lipid systems. Among others, this may concern new generations of mRNA vaccines or micelle-based carrier systems. From a more applied perspective, our study provides a blueprint for a quantitative analysis of any, not exclusively, polymer–lipid system by a combination of liquid chromatography, mass spectrometric, molecular hydrodynamic, light scattering, and electron microscopy techniques. Finally, our study emphasizes that a multimethod assessment of chemistry and structure provides a further foundation for a molecular understanding of the materials. Also, such analysis will support establishment of specifications of polymers required in European and U.S. pharmacopeias and compliance with good manufacturing practice standards.

## Supplementary Material



## Notes

Synthetic routes, additional experimental results and data evaluation from LC, MALDI-TOF MS, densimetry, sedimentation velocity AUC experiments, cryo-TEM images, and compiled hydrodynamic characteristics and properties (PDF)
