# Supplementary material for: POx-Lipids as an Alternative to PEG-Lipids? Multimethod Assessment of Chemistry and Structure
Source: Anal Chem. 2026 Mar 13;98(11):8277–88. doi: 10.1021/acs.analchem.5c07351 (PMC13019425; doi:10.1021/acs.analchem.5c07351)
Supplement: Supplementary file 1 [file ac5c07351_si_001.pdf]

SUPPORTING INFORMATION

POx-lipids as an alternative to PEG-lipids? Multimethod assessment of chemistry and structure

Ekaterina Tsarenko [a, b, ‡], *Ilya Anufriev* [a, c, ‡], *Caroline T. Holick* [a, b], *Tobias Klein* [a, b], *Stephanie Schubert* [a, b], *Nicole Fritz* [a, b], *Stephanie Hoeppener* [a, b], *Ulrich S. Schubert,* [a, b, c, d], *and Ivo Nischang* [a, b, c, d] *

[a]Laboratory of Organic and Macromolecular Chemistry (IOMC), Friedrich Schiller University Jena, Humboldtstr. 10, 07743 Jena, Germany

[b]Jena Center for Soft Matter (JCSM), Friedrich Schiller University Jena, Philosophenweg 7, 07743 Jena, Germany

[c]Helmholtz Institute for Polymers in Energy Applications Jena (HIPOLE Jena), Lessingstr. 12-14, 07743, Jena, Germany

[d]Helmholtz-Zentrum Berlin für Materialien und Energie GmbH (HZB), Hahn-Meitner-Platz 1, 14109 Berlin, Germany

*Corresponding Author

Ivo Nischang − Laboratory of Organic and Macromolecular Chemistry (IOMC), Friedrich Schiller University Jena, 07743 Jena, Germany; Jena Center for Soft Matter (JCSM), Friedrich Schiller University Jena, 07743 Jena, Germany; orcid.org/0000-0001-6182-5215; Phone: +49-3641-948-983; Email: ivo.nischang@uni-jena.de

Content

1. Materials, synthesis procedures, and preliminary characterization S3

1.1. Materials S3

1.2. Methods for preliminary characterization of the synthesized polymer-lipid conjugates: ^{1}H NMR, SEC, MALDI-TOF MS S3

1.3. Syntheses and preliminary characterization of the PEtOx with lipid end groups ($PEtOx_n$-lipid) S4

2. Additional method development, results and discussion S13

2.1. LC method development: column and eluent selection S13

2.2. ESI mass spectra of lipid S16

2.3. MALDI-TOF MS of $PEtOx_n$-lipid series S17

2.4. MALDI-TOF MS of commercial PEG-lipids S21

2.5. LC of $PEtOx_n$-lipid series and semi-quantitative purity determination S22

2.6. LC and MALDI-TOF MS of the $PEtOx_{46}$-$lipid_{clean}$ S24

2.7. Hydrodynamic characterization S26

2.7.1. Additional hydrodynamic relations S26

2.7.2. Hydrodynamic characterization of $PEtOx_n$-lipid series S28

2.7.3. Hydrodynamic characterization of $PEtOx_{46}$-lipids S34

3. References S37

1. Materials, synthesis procedures, and preliminary characterization

1.1. Materials

2-Ethyl-2-oxazoline (EtOx, ≥ 99%, Sigma-Aldrich) was pre-dried over barium oxide (BaO, 90%; Acros) and distilled under inert conditions. Methyl tosylate (MeOTs, 97%, Sigma-Aldrich) was dried over calcium hydride (CaH_2, Sigma-Aldrich) and distilled under reduced pressure. Acetic acid (AcOH, ACS Reag. Ph. Eur., VWR), triethylamine (NEt_3, Sigma-Aldrich), 0.5 M sodium methoxide (NaOMe) solution in methanol (≥ 99%, Sigma-Aldrich), *N*-hydroxysuccinimide (NHS, Sigma-Aldrich), 1-ethyl-3-(3-dimethylaminopropyl)carbodiimide (EDC, ≥ 97%, Sigma-Aldrich), ditetradecylamine (DTDA, 95%, Ambeed), 4-*N,N*-dimethylaminopyridine (DMAP, 99%, abcr), succinic anhydride (Sigma-Aldrich,) and chloroform ($CHCl_3$, anhydrous, > 99%, Sigma-Aldrich) were used without further purification. Acetonitrile, methanol, and *N,N*-dimethylformamide (DMF) were dried in a solvent purification system (SPS 800; MBRAUN). Technical grade diethyl ether (Et_2O) was used without further purification. A Spectra/Por®7 Dialysis Membrane (RC MWCO 1 kDa) was purchased from Carl Roth. Ditetradecylamine lacking tetradecylamine impurities was a kind gift from Evonik.

1.2. Methods for preliminary characterization of the synthesized polymer-lipid conjugates: ^1H NMR, SEC, MALDI-TOF MS

Proton nuclear magnetic resonance (^1H NMR) spectra were measured using a Bruker AC 300 MHz spectrometer or on a Bruker Avance IV NEO 500 MHz spectrometer equipped with a Prodigy BBO probehead and an automatic sample loading system (SampleCasePlus) for high-through-put sample analysis. The measurement was performed at room temperature using $CDCl_3$ as a solvent. The residual non-deuterated solvent signal was used for chemical shift referencing. The spectra were baseline corrected using the software SpinWorks 4.2.4.

Size exclusion chromatography (SEC) was measured in *N,N*-dimethylacetamide (DMAc) with 0.21 wt% LiCl (flow rate 1 mL min^{-1}) on an Agilent 1200 series system equipped with a PSS degasser, a G1310A pump, a G1329A autosampler, a Techlab oven at 40 °C, a G7162A refractive index detector (RID) and a PSS GRAM guard/30/1000 Å column set (10 µm particle

size, Polymer Standards Service (PSS) GmbH, Mainz, Germany). Polystyrene (PS) standards (PSS, 400 to 1,000,000 g mol^{-1}) were used for molar mass calibration.

Matrix-assisted laser desorption ionization time-of-flight mass spectrometry (MALDI-TOF MS) measurements were carried out utilizing a rapifleX MALDI TOF / TOF instrument (Bruker Daltonik, Bremen, Germany) equipped with scoutMTP II ion source and a smartbeam™ 3D laser ($\lambda = 355\,nm$) in positive reflector mode. *Trans*-2-[3-(4-*tert*-butylphenyl)-2-methyl-2-propenylidene] malononitrile (DCTB) was used as a matrix supplemented with sodium trifluoroacetate (NaTFA) or sodium iodide (NaI). DCTB was prepared at a concentration of 30 mg mL^{-1} in $CHCl_3$ or 25 mg mL^{-1} in THF. NaTFA was used at a concentration of 13.6 mg mL^{-1} in THF. PMMA calibration standards (2500 and 5000 Da) were prepared at a concentration 10 mg mL^{-1} in $CHCl_3$. For spotting on the target, PMMA standard mixtures were established in a ratio of 15/5/2.5 µL for DCTB/PMMA/NaTFA. Preparation of the fleXstandard solution (300 to 10000 Da) was done according to Bruker specifications and a mixture of 20/2.5/2.5 µL of DCTB/fleXstandard/NaTFA was used for spotting.

1.3. Syntheses and preliminary characterization of the PEtOx with lipid end groups (PEtOx$_n$-lipid)

The poly(2-ethyl-2-oxazoline)-lipids (PEtOx$_n$-lipids), where 2-ethyl-2-oxazoline (EtOx) is the monomer unit of polymers of certain degree of polymerization (DP) indicated by n, were synthesized through a previously reported four-step procedure (**Scheme S1**).[1] Briefly, EtOx was polymerized via cationic ring-opening polymerization (CROP). The CROP was terminated with acetic acid and triethylamine to introduce an acetate end-group yielding **PEtOx$_n$-OAc**. The end-group was then modified through successive post-polymerization reactions: A hydroxyl end group was generated by NaOMe catalyzed transesterification with methanol yielding **PEtOx$_n$-OH**. The alcohol was reacted with succinic anhydride to generate a carboxylic

acid end group yielding **PEtOx$_n$-COOH**. The latter was converted via amidation with ditetradecylamine to yield the final PEtOx with lipid end group **PEtOx$_n$-lipid**.

Scheme S1. Schematic representation of the synthesis route towards PEtOx with lipid end groups (**PEtOx$_n$-lipid**).

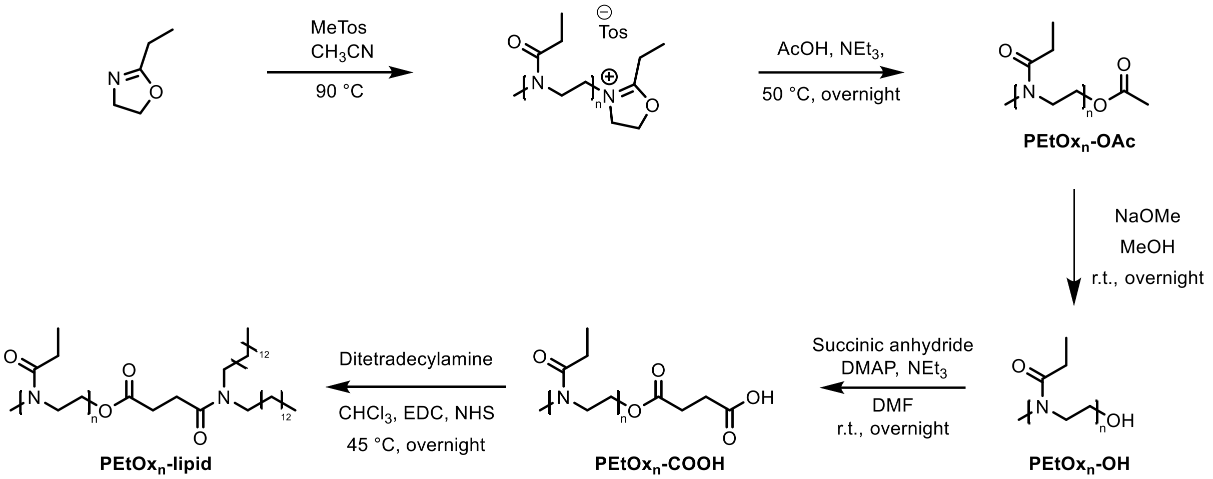

The synthesis and characterization data for **PEtOx$_n$-OAc**, **PEtOx$_n$-OH**, and **PEtOx$_{18}$-COOH**, **PEtOx$_{38}$-COOH**, **PEtOx$_{18}$-lipid**, **PEtOx$_{38}$-lipid**, and **PEtOx$_{46}$-lipid** have been published and described in detail before.[1] Synthesis and characterization data for **PEtOx$_{46}$-COOH**, **PEtOx$_{55}$-COOH**, **PEtOx$_{99}$-COOH**, **PEtOx$_{55}$-lipid, PEtOx$_{99}$-lipid**, and **PEtOx$_{46}$-lipid$_{clean}$** are described in the following.

PEtOx$_n$-COOH:

PEtOx$_n$-OH and DMAP were dissolved in anhydrous DMF. NEt_3 and succinic anhydride were added to the mixture, which was stirred overnight at room temperature. The polymer was then precipitated from diethyl ether (-80 °C) and redissolved in CH_2Cl_2. Subsequently, the mixture was washed with sat. aq. NH_4Cl solution and the organic phase was dried over $MgSO_4$. Subsequent to filtration, the volatiles were removed under reduced pressure. The residue was

redissolved in CH_2Cl_2 and precipitated from diethyl ether (-80 °C). The obtained solid was dried *in vacuo* overnight. The detailed amounts of substances used in each synthesis can be found in **Table S1**.

Table S1. Detailed amounts used for the synthesis of **PEtOx$_n$-COOH**.

Sample	PEtOx$_n$-OH [g|mmol|eq.]	Succinic anhydride [g|mmol|eq.]	DMF [mL]	DMAP [g|mmol| eq.]	NEt$_3$ [mg|mmol| eq.]
**PEtOx$_{46}$-COOH**	2.15|0.43|1	0.13|1.29|3	6	0.06|0.45|1.1	4.36|0.04|0.1
**PEtOx$_{55}$-COOH**	4.5|0.82|1	0.25|2.46|3	11	0.11|0.86|1.1	8.30|0.08|0.1
**PEtOx$_{99}$-COOH**	2.83|0.29|1	0.12|1.15|4	4	0.04|0.30|1.1	2.91|0.03|0.1

All **PEtOx$_n$-COOH** were analyzed by means of ^{1}H NMR spectroscopy, SEC, and MALDI-TOF MS. Data are listed below and displayed in **Figures S4** and **S5**.

PEtOx$_{46}$-COOH (3.46 g, 75%):

^{1}H NMR (500 MHz, $CDCl_3$): δ = 4.19 – 4.32 (br), 3.27 – 3.70 (br), 3.01 – 3.08 (m), 2.52 – 2.66 (m), 2.19 – 2.51 (m), 1.03 – 1.18 (br) ppm.

SEC (DMAc + 0.21 wt%, RI det. PS cal.) M_n = 9300 g mol^{-1}, Đ = 1.05.

MALDI (DCTB + NaTFA): $[CH_3(C_5H_9NO)_{42}C_4H_5O_4 + Na]^+$, *m/z* = 4315.90

PEtOx$_{55}$-COOH (3.36 g, 69%):

^{1}H NMR (500 MHz, $CDCl_3$): δ = 4.18 – 4.31 (br), 3.25 – 3.67 (br), 2.98 – 3.07 (m), 2.49 – 2.67 (m), 2.17 – 2.49 (m), 1.01 – 1.18 (br) ppm.

SEC (DMAc + 0.21 wt%, RI det. PS cal.) M_n = 10500 g mol^{-1}, Đ = 1.06.

MALDI (DCTB + NaTFA): $[CH_3(C_5H_9NO)_{49}C_4H_5O_4 + Na]^+$, *m/z* = 5009.38

PEtOx$_{99}$-COOH (2.39 g, 84%):

^{1}H NMR (500 MHz, $CDCl_3$): δ = 4.18 – 4.31 (br), 3.19 – 3.74 (br), 3.00 – 3.07 (m), 2.49 – 2.71 (m), 2.18 – 2.50 (m), 1.02 – 1.18 (br) ppm.

SEC (DMAc + 0.21 wt%, RI det. PS cal.) M_n = 16800 g mol^{-1}, Đ = 1.16.

MALDI (DCTB + NaTFA): $[CH_3(C_5H_9NO)_{106}C_4H_5O_4 + Na]^+$, *m/z* = 10657.29.

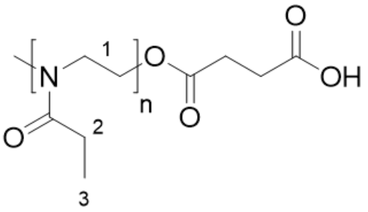

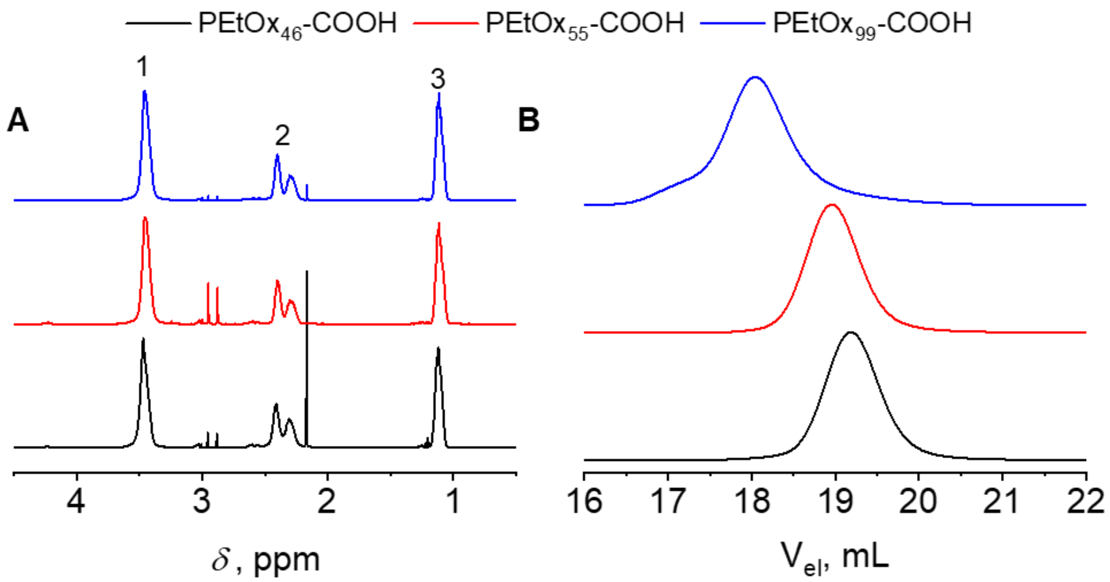

Figure S1. (**A**) ^{1}H NMR spectra of **PEtOxn-COOH** (300 or 500 MHz, $CDCl_3$) and peak assignment. (**B**) SEC elugrams of **PEtOxn-COOH** (RID, DMAc + 0.21 wt% LiCl).

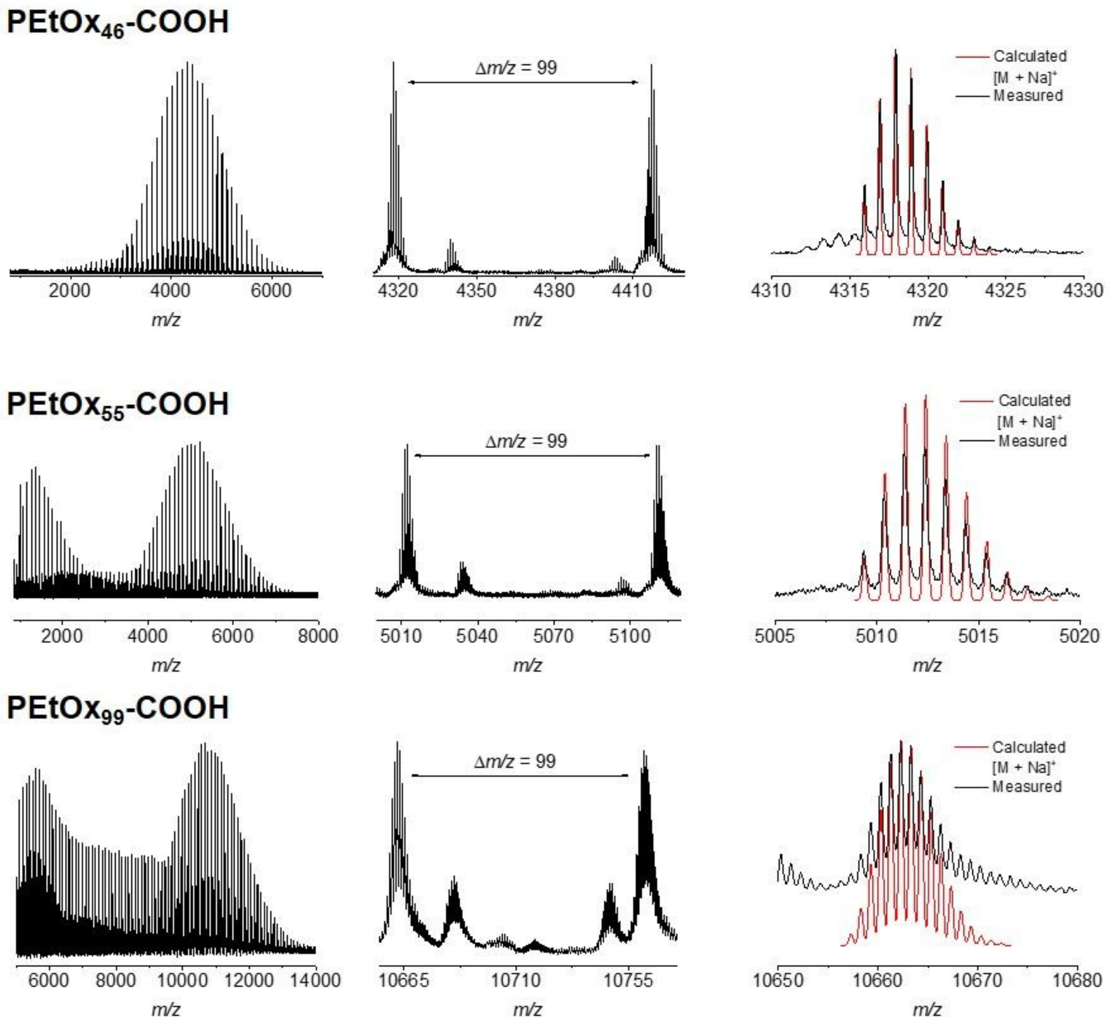

Figure S2. MALDI-TOF MS analysis of **PEtOx$_n$-COOH** (DCTB + NaTFA). From left to right: Full spectra, display of the repeating unit of PEtOx (Δ*m/z* = 99) and overlay of the measured and calculated isotopic patterns of the most abundant species (black: measured, red: calculated).

PEtOx$_n$-lipid:

PEtOx$_n$-COOH was dissolved in anhydrous $CHCl_3$ and DMAP, NHS, and EDC were added. The mixture was stirred for 3 h at room temperature. Ditetradecylamine was added and the solution was heated to 45 °C overnight. The mixture was precipitated into diethyl ether (-80

°C). The precipitate was redissolved in CH_2Cl_2 and cooled to −20 °C for 3 to 5 h. The precipitate was filtered off (0.25 µm PTFE filter). The soluble parts were precipitated in diethyl ether (-80 °C) and the precipitate was dialyzed (1000 Da MWCO dialysis membrane, 3 d against EtOH:water (1:1), 2 d against water), and freeze dried. The detailed amounts of substances used in each synthesis can be found in **Table S2**.

Table S2. Detailed amounts used for the synthesis of **PEtOx$_n$-lipid**.

Sample	PEtOx$_n$-COOH [g|mmol|eq.]	NHS [g|mmol|eq.]	DTDA [g|mmol|eq.]	EDC [g|mmol|eq.]	DMAP [mg|mmoleq.]	$CHCl_3$ [mL]
**PEtOx$_{55}$-lipid**	2.5|0.45|1	0.13|1.12|2.5	0.73|1.79|4	0.26|1.34|3	5.47|0.05|0.1	14
**PEtOx$_{99}$-lipid**	2.3|0.23|1	0.07|0.57|2.5	0.37|0.91|4	0.13|0.68|3	2.76|0.02|0.1	7

All **PEtOx$_n$-lipids** were analyzed by means of ^{1}H NMR spectroscopy, SEC, and MALDI-TOF MS. Data are listed below and displayed in **Figures S6** and **S7**.

PEtOx$_{55}$-lipid (2.00 g, 75%):

^{1}H NMR (500 MHz, $CDCl_3$): δ = 4.14 – 4.27 (br), 3.29 – 3.69 (br), 3.17 – 3.30 (m), 2.93 – 3.08 (m), 2.56 – 2.68 (m), 2.21 – 2.49 (m), 1.42 – 1.60 (m), 1.19 – 1.33 (br), 0.99 – 1.18 (br), 0.87 (t) ppm.

SEC (DMAc + 0.21 wt%, RI det. PS cal.) M_n = 11400 g mol^{-1}, Đ = 1.05.

MALDI (DCTB + NaTFA): $[CH_3(C_5H_9NO)_{47}C_4H_4O_3NC_{28}H_{58} + Na]^+$, *m/z* = 5202.70.

PEtOx$_{99}$-lipid (2.09 g, 89%):

^{1}H NMR (500 MHz, $CDCl_3$): δ = 4.16 – 4.27 (br), 3.29 – 3.69 (br), 3.17 – 3.29 (m), 2.93 – 3.08 (m), 2.57 – 2.72 (m), 2.18 – 2.58 (m), 1.40 – 1.62 (m), 1.20 – 1.34 (br), 1.03 – 1.20 (br), 0.87 (t) ppm.

SEC (DMAc + 0.21 wt%, RI det. PS cal.) M_n = 17600 g mol^{-1}, Đ = 1.11.

MALDI (DCTB + NaTFA): $[CH_3(C_5H_9NO)_{100}C_4H_4O_3NC_{28}H_{58} + Na]^+$, *m/z* = 10453.64.

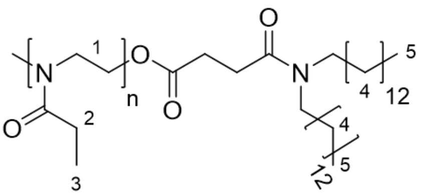

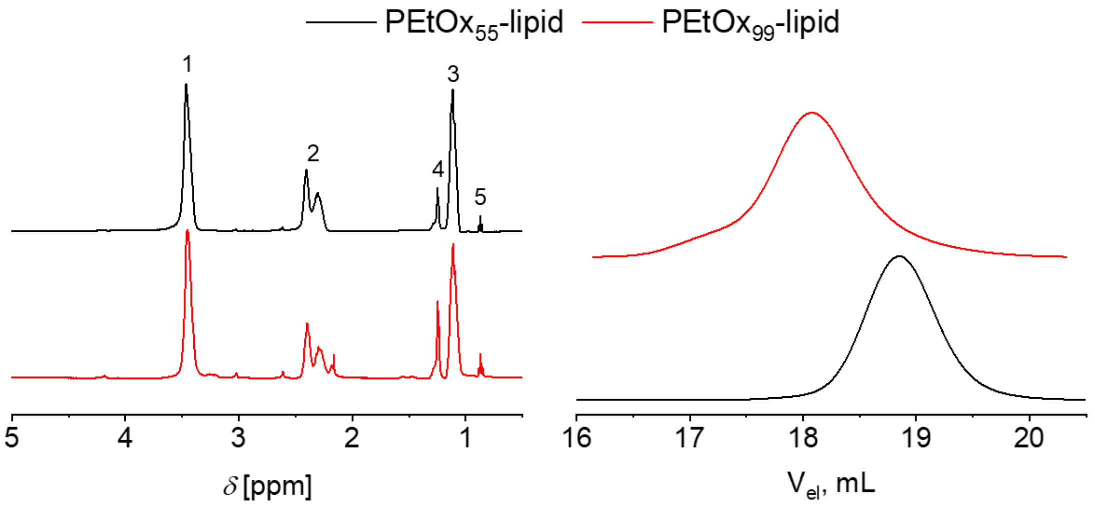

Figure S3. (**A**) ^{1}H NMR spectra of **PEtOx$_n$-lipid** (300 or 500 MHz, $CDCl_3$) and peak assignment. (**B**) SEC elugrams of **PEtOx$_n$-lipid** (RID, DMAc + 0.21 wt% LiCl).

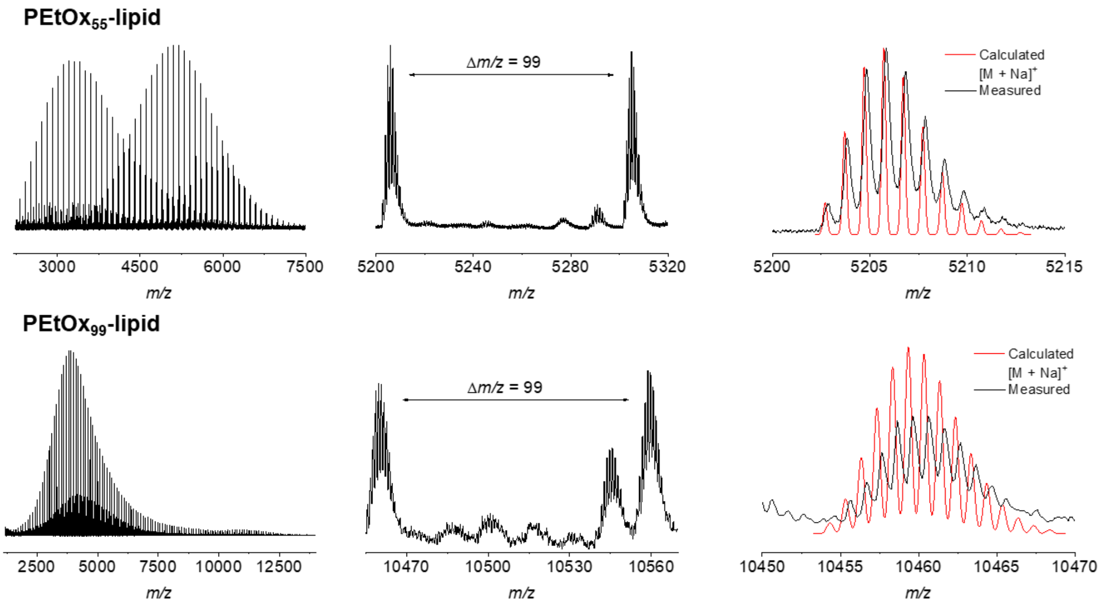

Figure S4. MALDI-TOF MS analysis of **PEtOx$_n$-lipid** (DCTB + NaTFA). From left to right: Full spectra, display of the repeating unit of EtOx (Δ*m/z* = 99), and overlay of the measured and calculated isotopic patterns of the most abundant species (black: measured, red: calculated).

PEtOx$_{46}$-lipid$_{clean}$:

PEtOx$_{46}$-lipid$_{clean}$ was synthesized in a similar fashion using ditetradecylamine lacking tetradecylamine impurities. **PEtOx$_{46}$-COOH** (131 mg, 0.03 mmol, 1 eq.), DMAP (0.3 mg, 0.002 mmol, 0.1 eq.), NHS (8.4 mg, 0.07 mmol, 2.5 eq.), and EDC (14.8 µL, 0.08 mmol, 3 eq.) were dissolved in $CHCl_3$ (850 µL). After stirring for three hours at room temperature, ditetradecylamine (23 mg, 0.06 mmol, 2 eq.) was added to the mixture, which was then stirred at 45 °C overnight. The mixture was subsequently diluted with $CHCl_3$, washed twice with aq. $NaHCO_3$ solution, and once with brine. The organic phase was dried over Na_2SO_4, filtered, and the solvent was removed under reduced pressure. The polymer was dissolved in CH_2Cl_2, precipitated in diethyl ether (-80 °C), and the pellet was dried *in vacuo*. The product was obtained as a colorless powder (yield: 112.4 mg, 86%).

PEtOx$_{46}$-lipid$_{clean}$ was analyzed by means of ^{1}H NMR spectroscopy, SEC, and MALDI-TOF MS. Data are listed below and displayed in **Figure S8**.

^{1}H NMR (300 MHz, $CDCl_3$): δ = 4.28 – 4.14 (br.), 3.64 – 3.32 (br.), 3.31 – 3.14 (br.), 3.09 - 2.92 (m), 2.69 – 2.56 (br.), 2.55 – 2.13 (m), 1.65 – 1.40 (m), 1.38 – 1.22 (s), 1.20 – 1.00 (br.), 0.97 – 0.80 (br.) ppm.

SEC (DMAc + 0.21 wt%, RI det. PS cal.) M_n = 10700 g mol^{-1}, Đ = 1.03.

MALDI-TOF MS (DCTB + NaTFA): $[CH_3(C_5H_9NO)_{38}C_4H_4O_3NC_{28}H_{58} + Na]^+$ *m/z* = 4311.07.

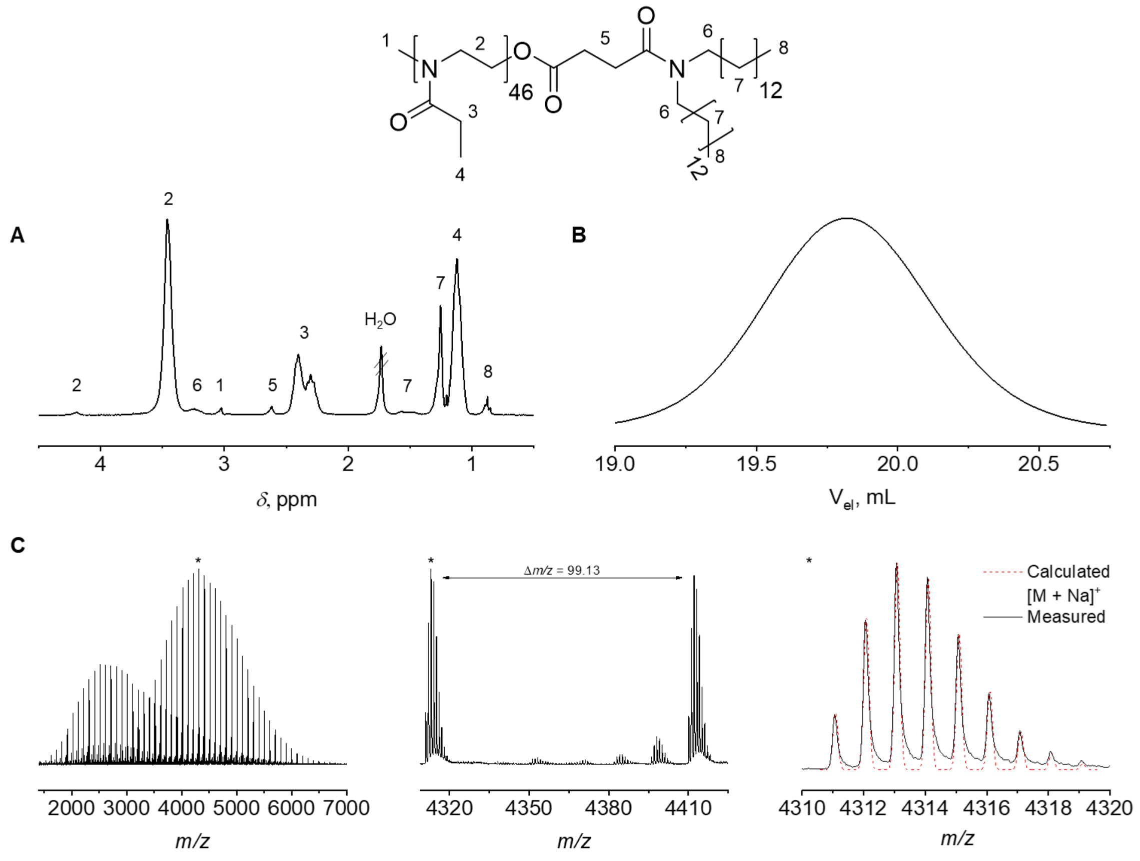

Figure S5. Characterization of **PEtOx$_{46}$-lipid$_{clean}$**. (**A**) ^{1}H NMR spectrum (300 MHz, $CDCl_3$) and assignment of the signals to the schematic representation of the structure. (**B**) SEC elugram (RID, DMAc + 0.21 wt% LiCl). (**C**) MALDI-TOF mass spectrum (DCTB + NaTFA). From left to right: Full spectrum, display of the repeating unit EtOx, and an overlay of the isotopic pattern of the most abundant species (*, black: measured, red: calculated).

2. Additional method development, results and discussion

2.1. LC method development: column and eluent selection

In scouting experiments, a Chromolith® Performance RP-8 endcapped and a Chromolith® HighResolution RP-18 endcapped monolithic silica column from Merck KGaA (Darmstadt, Germany) were used. Both columns had a nominal length of 100 mm and an internal diameter of 4.6 mm. Acetonitrile/H_2O or acetonitrile/0.1% (v/v) formic acid (FA) were used as mobile phase. In first experiments, simple linear gradient elution was utilized. The acetonitrile content in the mobile phase was increased in a linear fashion from 20 to 98% (%, v/v) in 9 min. Here, the eluent composition was kept constant at 98% of acetonitrile for 20 min. Afterward, the acetonitrile content was decreased to the initial 20% and the column re-equilibrated for 5 min before the next injection.

In preliminary experiments, the selection of mobile and stationary phase for the analysis of the polymer-lipid conjugates was performed. For that, ditetradecylamin (lipid) and $PEtOx_{18}$-lipid were used as examples (**Figure S6**). Two reversed-phase monolithic silica columns bonded with alkyl chains of different length, i.e., octyl (C8) and octadecyl (C18), were tested. The elution was performed using a linear gradient of acetonitrile in water (H_2O/acetonitrile) or in 0.1% (v/v) aqueous formic acid (FA/acetonitrile) at otherwise identical chromatographic elution conditions.

The secondary amine with two tetradecyl alkyl chains showed strong retention on a reversed phase column due to hydrophobic interactions with the bonded alkyl chains of the stationary phase. Utilizing an acidified eluent led to protonation of the amino moiety of the lipid and its faster elution from the column due to its increased hydrophilicity (**Figure S6C** and **S6D**). The elugram of the $PEtOx_{18}$-lipid showed several populations which contain the polymer main fraction, free lipid as well as non-conjugated $PEtOx_{18}$-OH (**Figure S6E – S6H**). Utilizing the column with longer (C18) alkyl chains led to incomplete elution of the hydrophobic polymer-lipid species (**Figure S6E** and **S6F** when compared to **Figure S6G** and **S6H**).

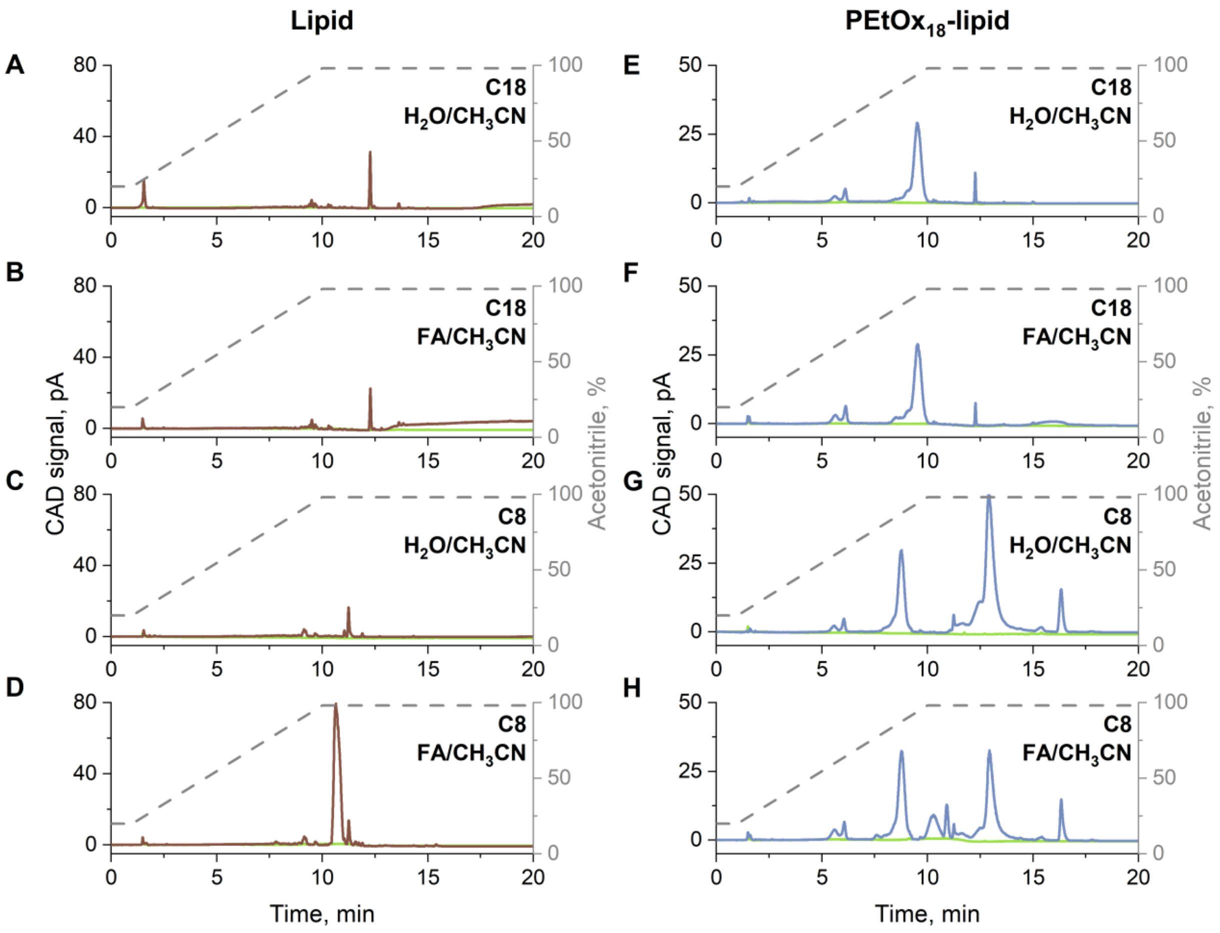

Figure S6. Method development for PEtOx$_n$-lipid elution. In preliminary experiments, gradient elution of (**A – D**) ditetradecylamine (lipid) and PEtOx$_{18}$-lipid (**E – H**) was monitored by CAD. Selection of the stationary phase (Chromolith® Performance RP-8 endcapped (C8) or Chromolith® HighResolution RP-18 endcapped (C18)) as well as mobile phase composition (water/acetonitrile (H_2O/acetonitrile) or 0.1% (v/v) aqueous FA/acetonitrile) varied. Measurement conditions: linear gradient elution (as indicated with a gray dashed line), column temperature 35 °C, flow rate 1 mL min^{-1}, CAD.

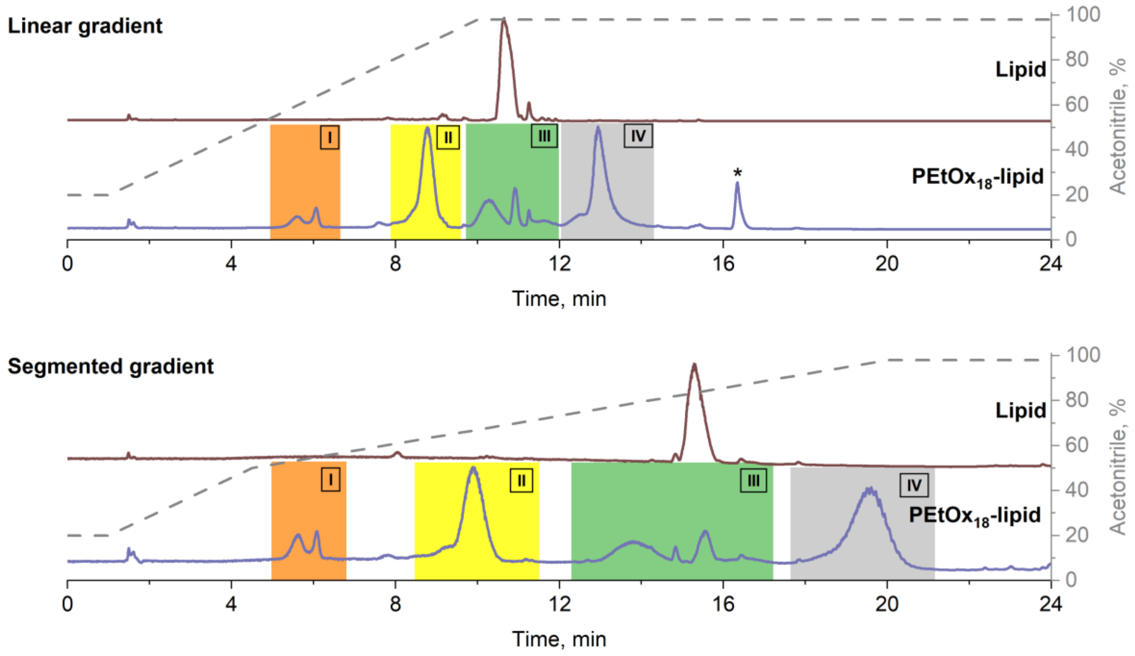

Figure S7. Final LC method establishment: from linear to segmented gradient elution. Normalized elugrams of ditetradecylamine (lipid) and $PEtOx_{18}$-lipid. Measurement conditions: Chromolith® Performance RP-8 endcapped (C8) column, eluent 0.1% (v/v) aqueous formic acid/acetonitrile (FA/acetonitrile), gradient elution (as indicated with a gray dashed line), column temperature 35 °C, flow rate 1 mL min^{-1}, CAD. *This signal for the linear gradient was common to all samples and appeared after the eluent contained acetonitrile only for more than 5 min. It was visible after 25-26 min in the segmented gradient.

2.2. ESI mass spectra of lipid

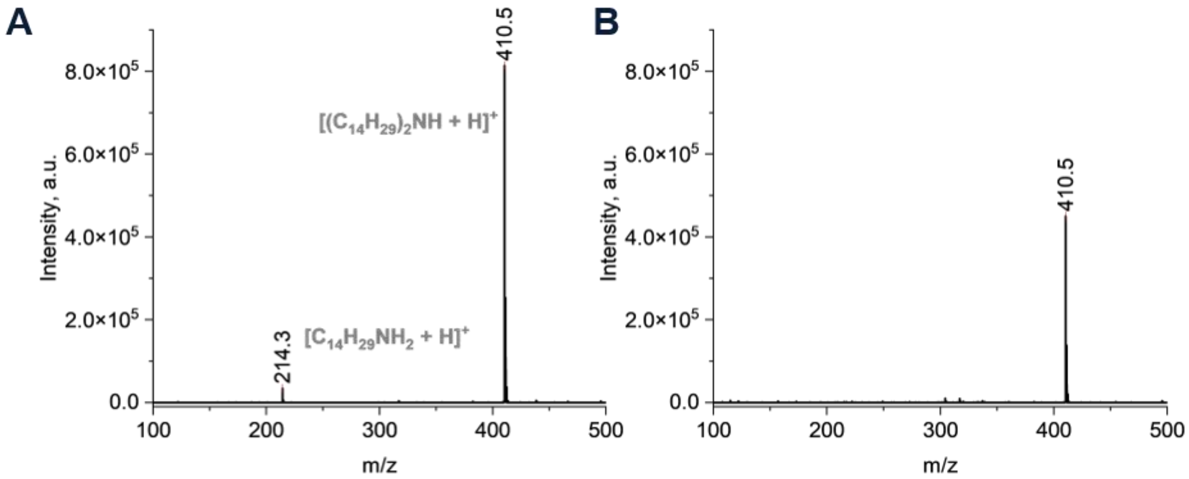

Figure S8. ESI mass spectra of the lipid reagent of two different purity levels: (**A**) ditetradecylamine (>95% signal intensity) with tetradecylamine impurity and (**B**) ditetradecylamine (100% signal intensity).

2.3. MALDI-TOF MS of PEtOx$_n$-lipid series

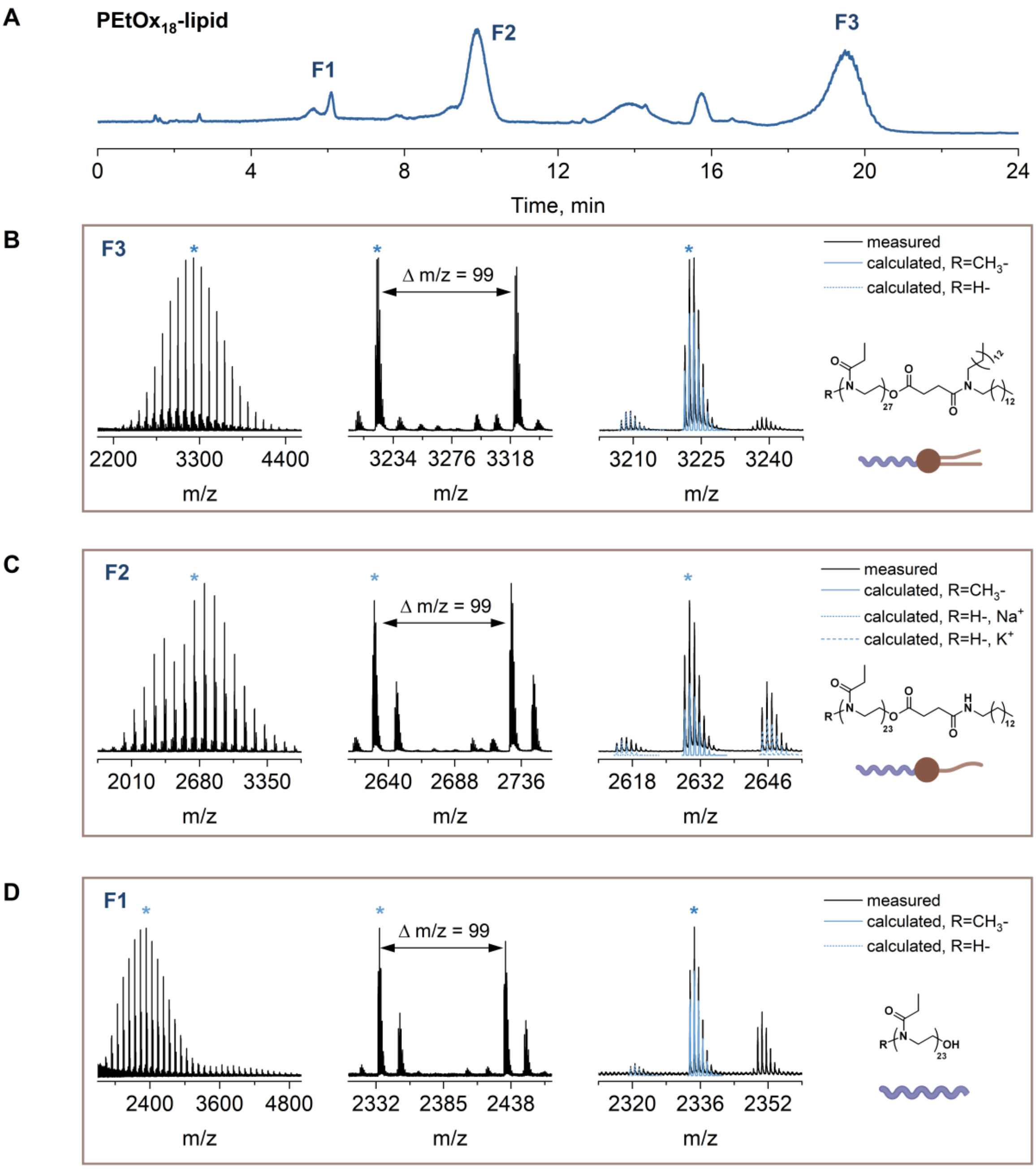

Figure S9. Composition analysis of PEtOx$_{18}$-lipid by liquid chromatography offline coupled to MALDI-TOF MS.

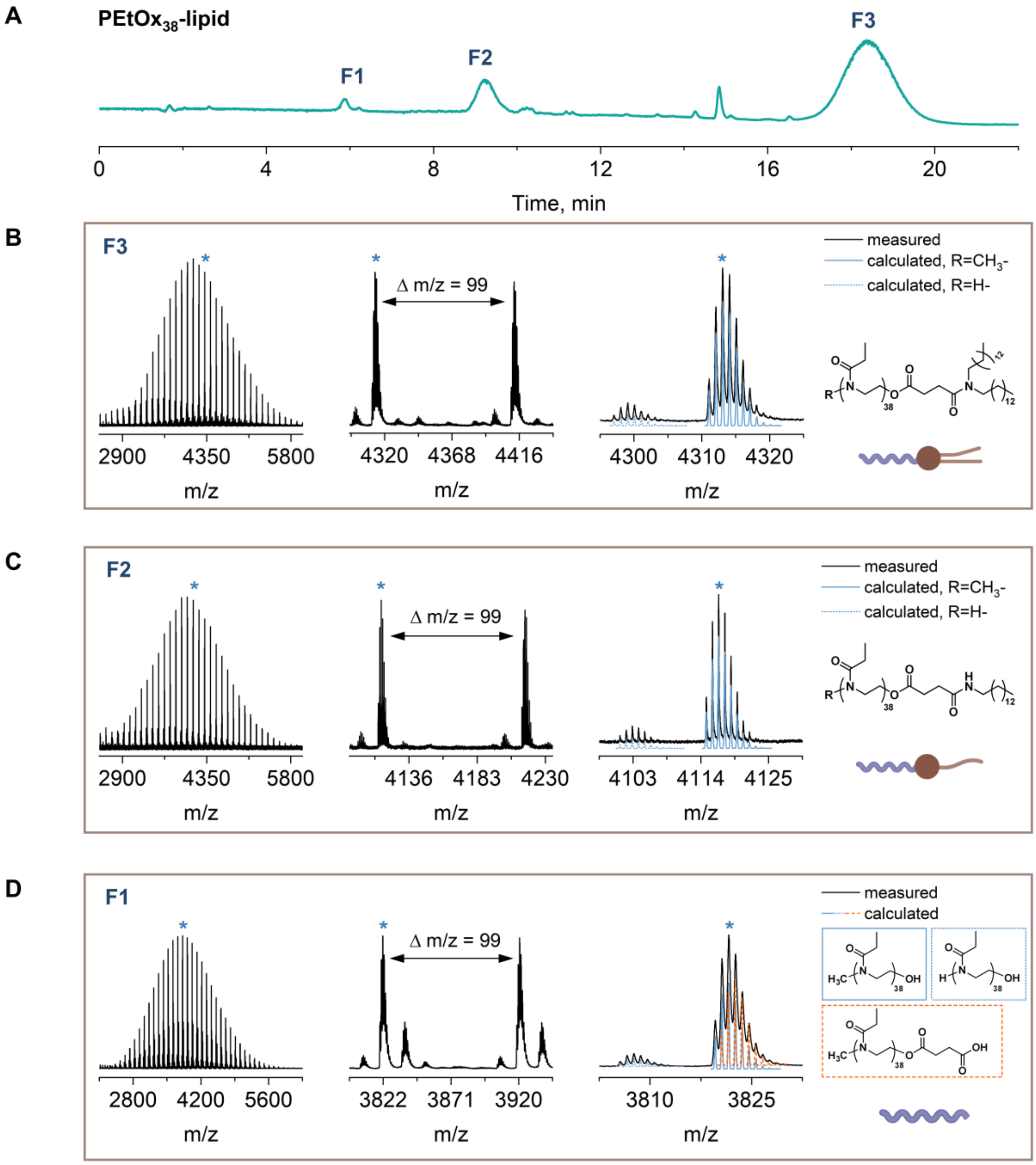

Figure S10. Composition analysis of PEtOx38-lipid by liquid chromatography offline coupled to MALDI-TOF MS.

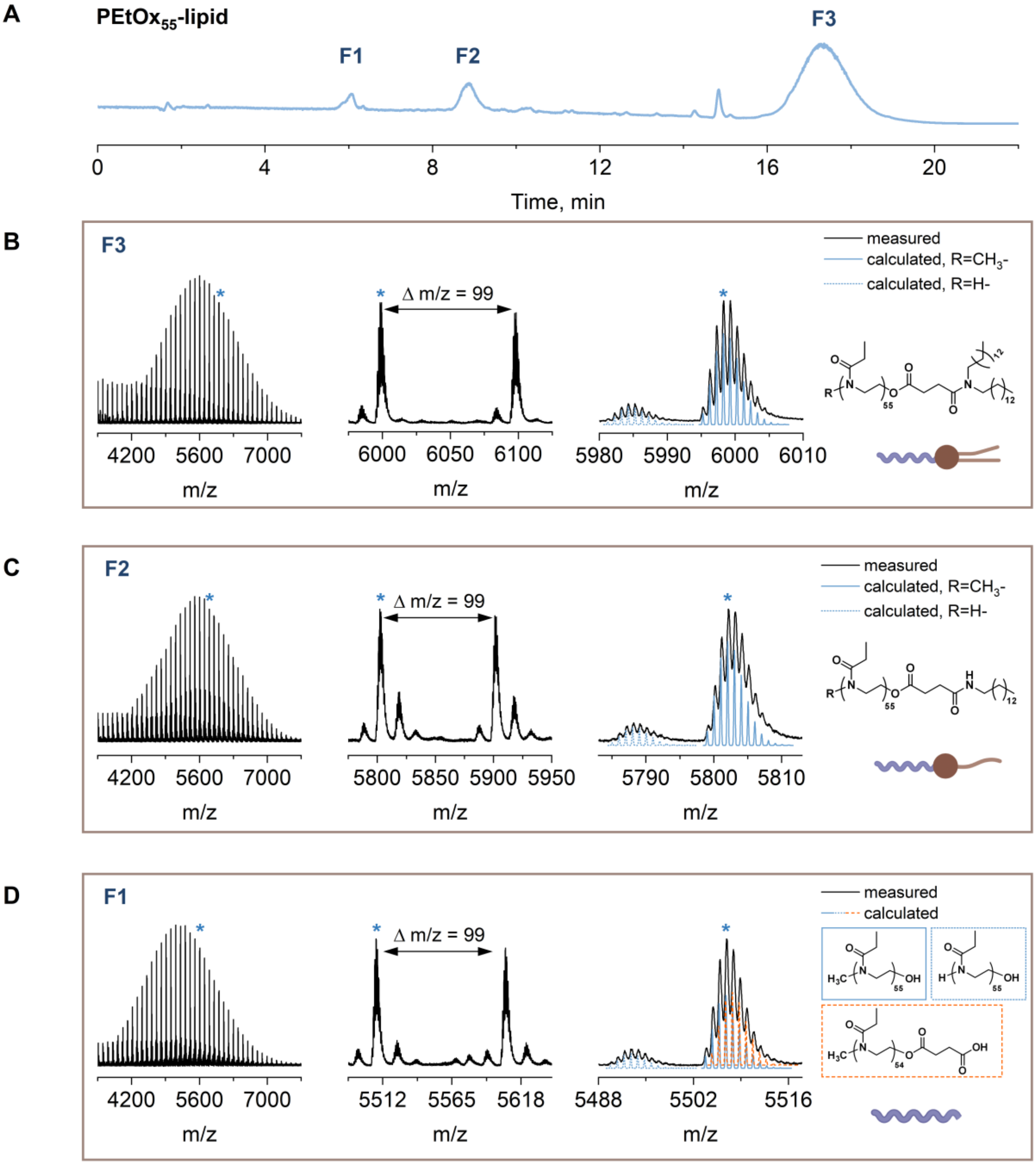

Figure S11. Composition analysis of PEtOx55-lipid by liquid chromatography offline coupled to MALDI-TOF MS.

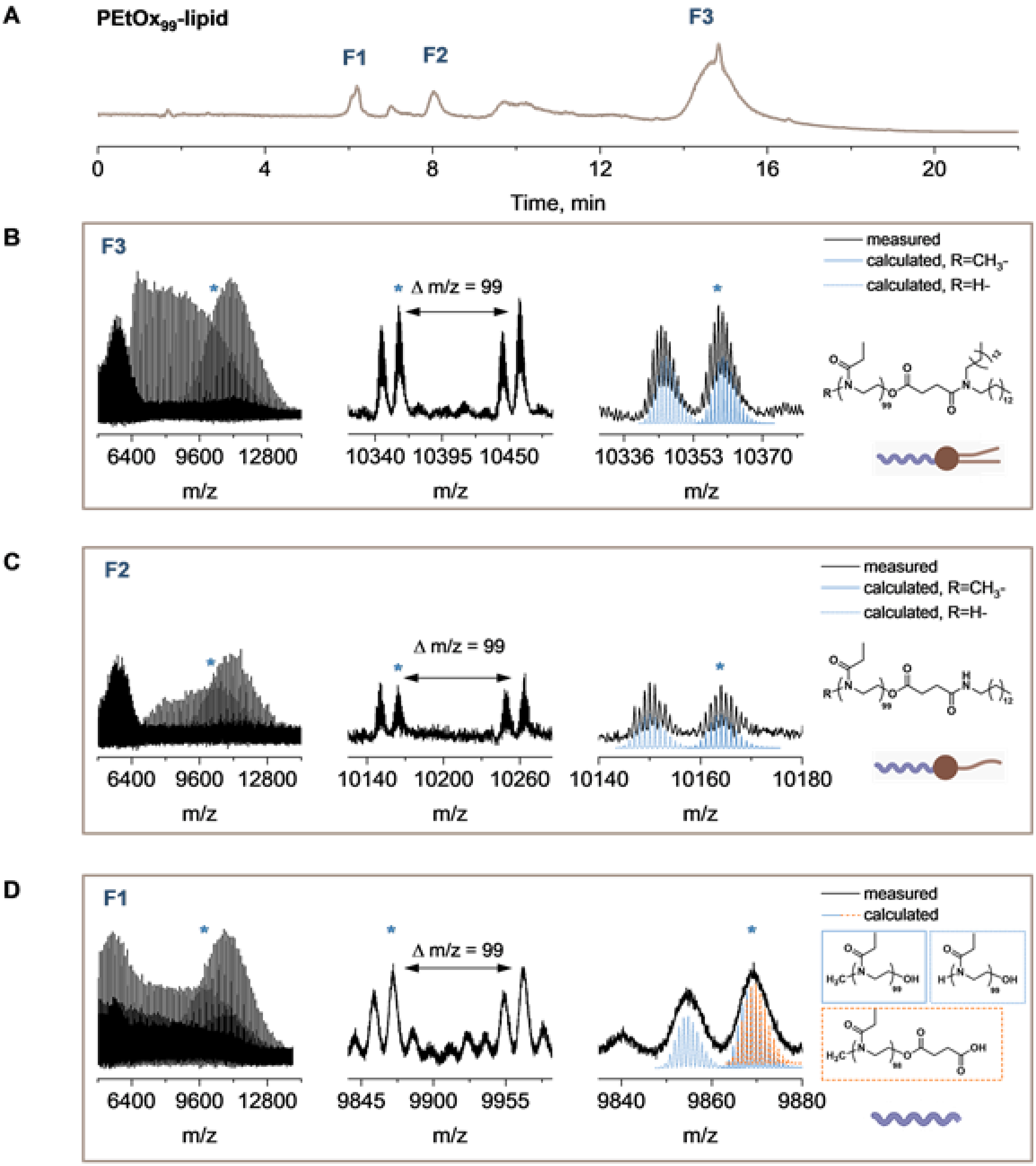

Figure S12. Composition analysis of PEtOx99-lipid by liquid chromatography offline coupled to MALDI-TOF MS.

2.4. MALDI-TOF MS of commercial PEG-lipids

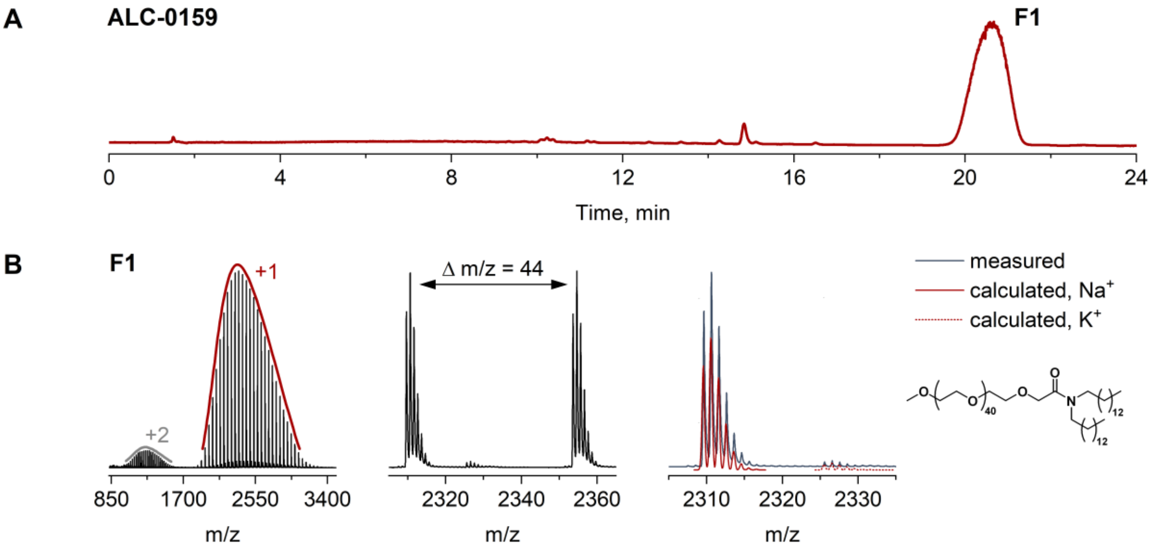

Figure S13. Composition analysis of commercial PEG-lipid (ALC-0159) by liquid chromatography offline coupled to MALDI-TOF MS.

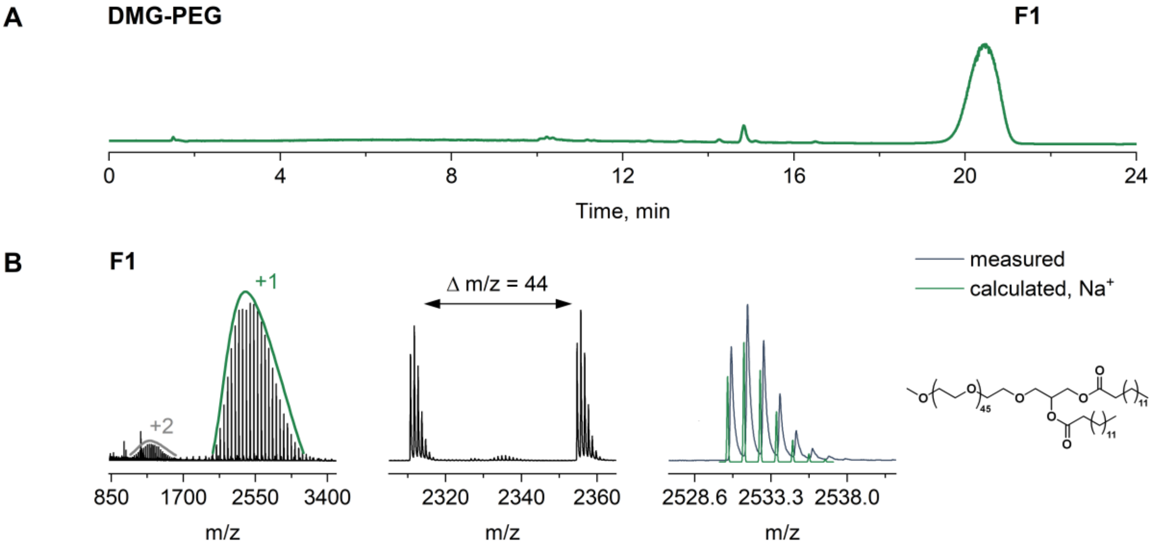

Figure S14. Composition analysis of commercial PEG-lipid (DMG-PEG) by liquid chromatography offline coupled to MALDI-TOF MS.

2.5. LC of $PEtOx_n$-lipid series and semi-quantitative purity determination

Table S3. Purity of the $PEtOx_n$-lipids with different degree of polymerization (DP) of EtOx monomers indicated by n, determined as relative peak area (%) of the pure fraction in the elugrams (**Figure 1**).

Sample	Purity, %
$PEtOx_{18}$-lipid	43
$PEtOx_{38}$-lipid	82
$PEtOx_{46}$-lipid	85
$PEtOx_{55}$-lipid	85
$PEtOx_{99}$-lipid	63

Table S4. Purity of the PEtOx46-lipid determined as relative peak area (%) values of the pure fraction in the elugrams (**Figure S15**).

Concentration, mg mL^{-1}	Purity, %	Total purity, %
0.50	85.50	85.2 ± 0.4
0.75	85.51	
1.00	85.17	
1.30	84.54	

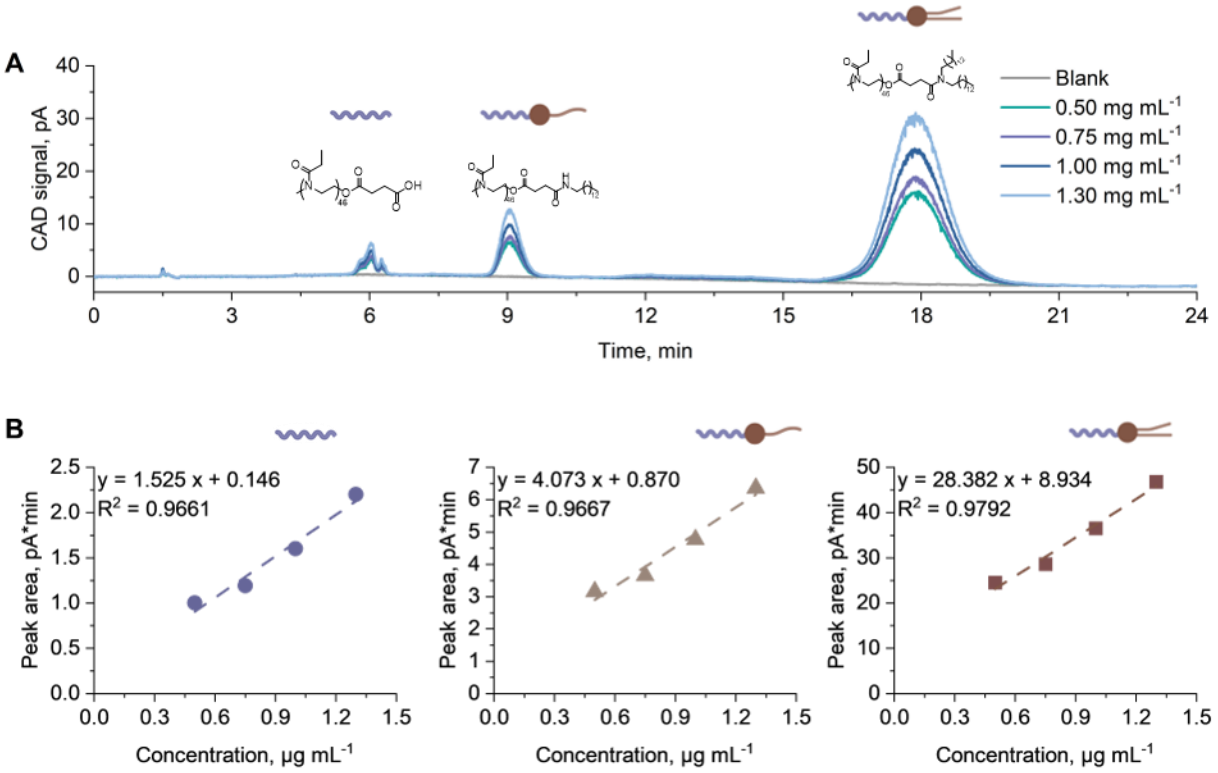

Figure S15. Purity determination for PEtOx46-lipid. (**A**) Overlay of elugrams of PEtOx46-lipid dissolved in methanol at different concentrations as indicated in the graph. Measurement conditions are specified in the experimental section. The signals in the elugrams refer to PEtOx precursor (PEtOx46-OH and PEtOx46-COOH, $t_R \approx 6$ min), PEtOx functionalized with tetradecylamine ($t_R \approx 9$ min) and PEtOx functionalized with ditetradecylamine ($t_R \approx 18$ min), respectively. (**B**) Peak area for indicated fractions plotted as a function of concentration. Linear fitting of the data was implemented to indicate linearity of CAD response in the narrow concentration range.

2.6. LC and MALDI-TOF MS of the PEtOx$_{46}$-lipid$_{clean}$

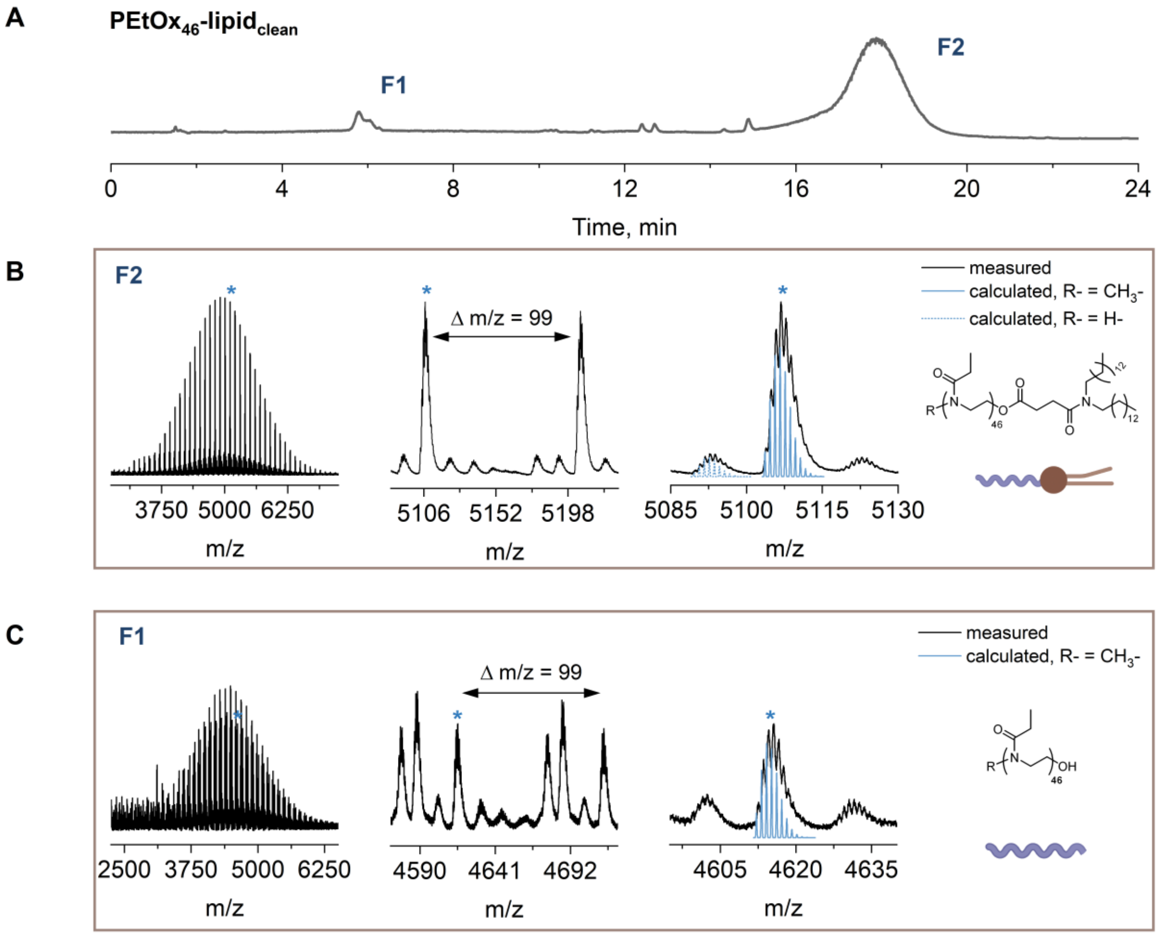

Figure S16. Composition analysis of PEtOx$_{46}$-lipid$_{clean}$ by liquid chromatography offline coupled to MALDI-TOF MS.

Table S5. Purity of the PEtOx46-lipidclean determined as relative peak area (%) values of the pure fraction in the elugrams (**Figure 1**).

Concentration, mg mL-1	Purity, %	Total purity, %
0.50	92.73	92.4 ± 0.5
0.75	93.03	
1.00	92.20	
1.30	91.76	

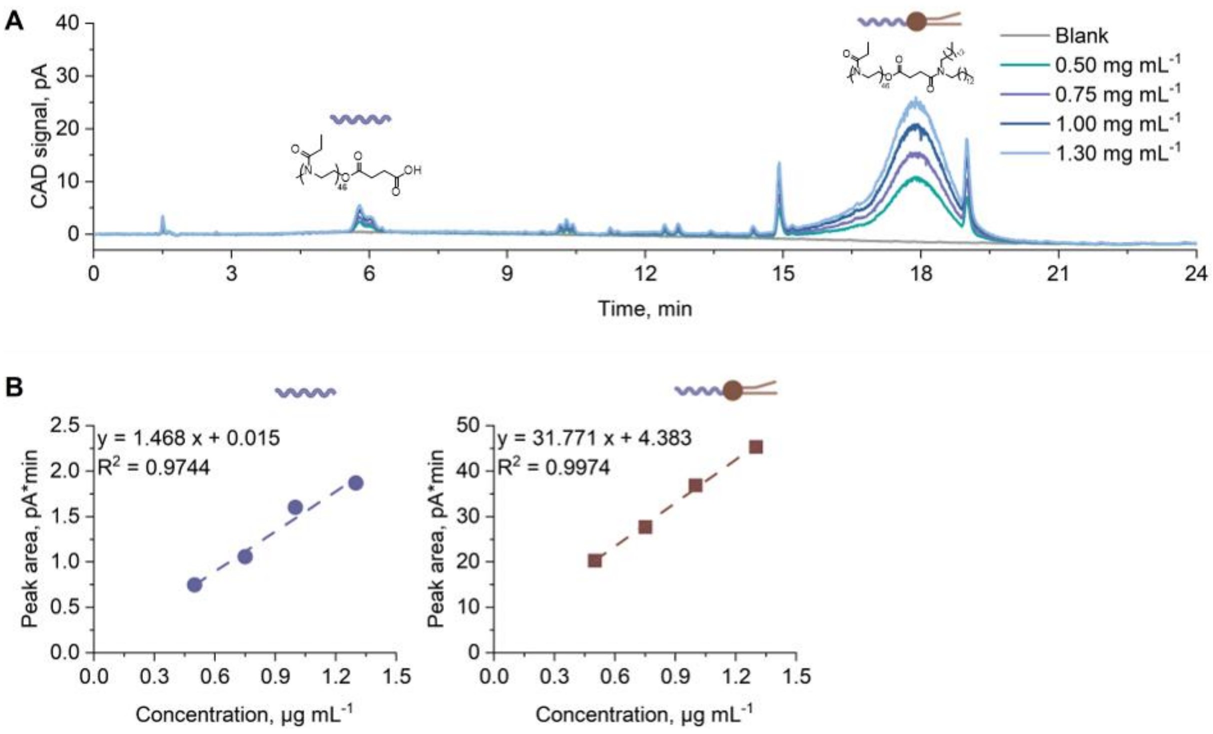

Figure S17. Purity determination for the PEtOx46-lipidclean. (**A**) Overlaid elugrams of PEtOx46-lipidclean dissolved in methanol at different concentrations as indicated in the graph. Measurement conditions are specified in the experimental section. The signals in the elugrams refer to PEtOx precursor (PEtOx46-OH and PEtOx46-COOH, $t_R \approx 6$ min) and PEtOx functionalized with ditetradecylamine ($t_R \approx 18$ min), respectively. The sharp peaks may stem from impurities in the vial. (**B**) Peak area for indicated fractions plotted as a function of concentration. Linear fitting of the data was implemented to indicate linearity of CAD response in the narrow concentration range.

2.7. Hydrodynamic characterization

2.7.1. Additional hydrodynamic relations

The intrinsic viscosities of the studied samples, $[\eta]$, were determined by the Huggins and Kraemer extrapolations:

$$\frac{\eta_r - 1}{c} = [\eta] + k_H[\eta]^2 c + \cdots (eq.S1)$$

$$\frac{\ln \eta_r}{c} = [\eta] + k_K[\eta]^2 c + \cdots (eq.S2)$$

where η_r is the relative viscosity, c is the sample concentration, k_H is the Huggins constant, k_K is Kraemer constant.

The sedimentation velocity experiments were analyzed by the numerical solution of the Lamm equation $(eq. 1)$. To determine the sedimentation coefficient of the polymer at infinite dilution, s_0, measurements were performed at different concentration and resulting values of s were extrapolated to infinite dilution via

$$s^{-1} = s_0^{-1}(1 + k_s c)\ (eq.\ S3)$$

where k_s is the concentration-sedimentation or Gralen coefficient.

A similar procedure was followed for the translational frictional ratios f/f_{sph}:

$$f/f_{sph} = (f/f_{sph})_0\big(1 + k_f c\big)\ (eq.\ S4)$$

where k_f is the concentration-frictional ratio coefficient.

In case there was no apparent concentration-dependent behavior, average values of s and f/f_{sph} from the different concentrations were utilized. The molar mass of studied samples, $M_{s,f}$, was determined via substitution of D from eq. 2 in the Svedberg equation:

$$M_{s,D} = \frac{sRT}{D(1 - \upsilon\rho_0)}\ (eq.S5)$$

Consequently, the modified Svedberg equation reads:

$$M_{s,f} = 9\pi\sqrt{2}N_A\left([s]\big(f/f_{sph}\big)_0\right)^{3/2}\sqrt{\bar{\upsilon}}\ (eq.S6)$$

where N_A is the Avogadro number, $[s]$ is the intrinsic sedimentation coefficient, and υ is the partial specific volume.

The hydrodynamic diameters, d_h, from SV-AUC experiments were calculated by substituting D from eq. 2 in the Stokes-Einstein equation:

$$D = \frac{kT}{f} = \frac{kT}{3\pi\eta_0 d_h} \ (eq. S7)$$

where k is the Boltzmann constant, T is the temperature, and $f = 6\pi\eta_0 d_h$ is the translational friction coefficient of a sphere. η_0 is the viscosity of the solvent.

Consequently, d_h values are defined by:

$$d_h = 3\sqrt{2}\sqrt{[s]\upsilon}\left(f/f_{sph}\right)_0^{3/2} \ (eq. S8).$$

2.7.2. Hydrodynamic characterization of PEtOx$_n$-lipid series

Figure S18 shows results from viscometric studies of the synthesized PEtOx$_n$-lipid systems in solvents water and ethanol. In ethanol (**Figure S18A** and **S18C**) systems show linear behavior of Huggins and Kraemer extrapolation plots (eq. S1 and S2) being typical for linear polymers in a reasonably good solvent. Resulting values of intrinsic viscosity, $[\eta]$, are in a range from 7.2 $cm^3 g^{-1}$(PEtOx$_{18}$-lipid) to 15.0 $cm^3 g^{-1}$ (PEtOx$_{99}$-lipid). In water, Huggins plots show nonlinear behavior (**Figure S18B** and **S18D**).[2] Furthermore, the nonlinearity in water increases with the increase of the polymer DP, perhaps due to the dynamic properties of the polymer micelles in water.

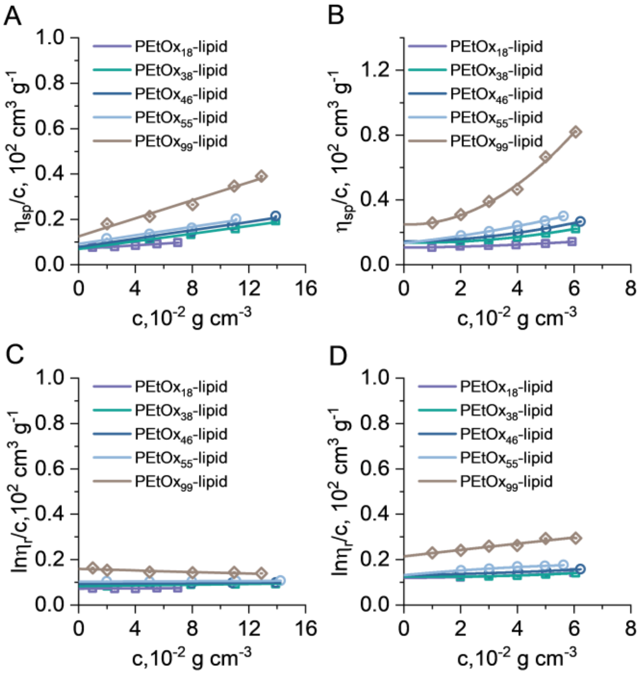

Figure S18. Huggins extrapolation plots for intrinsic viscosity estimations of PEtOx$_n$-lipids in (**A**) ethanol and (**B**) water as solvents. Kraemer extrapolation plots for intrinsic viscosity estimations in (**C**) ethanol and (**D**) water as solvents. For extrapolation plots in water, a quadratic extension of concentration for $[\eta]$-estimations was used.

The measurements of density increments (**Figure S19**) resulted in similar values of partial specific volume, υ, in both solvents (**Table S6**). As the DP increases, the partial specific volume, υ, in solvent water approach that of PEtOx obtained from a previous study ($\upsilon =$

0.84 cm^3g^{-1}).[3] This trend can be attributed to the decreasing impact of the lipid terminus on polymer chain properties (**Figure S20**).

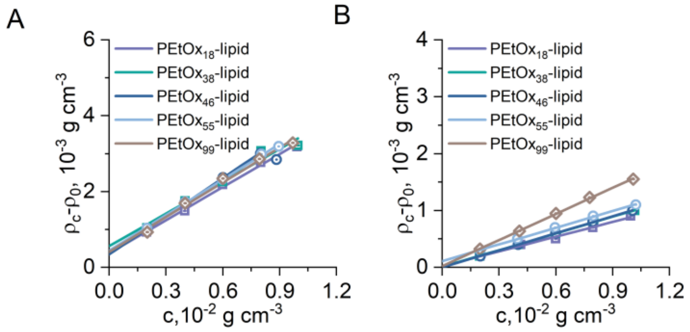

Figure S19. Concentration dependences of $\rho_c - \rho_0$ of PEtOx$_n$-lipids in (**A**) ethanol and (**B**) water as solvents.

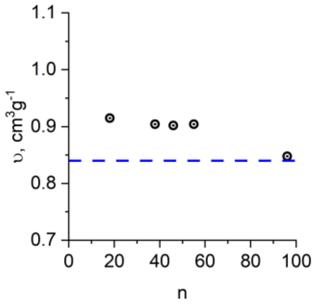

Figure S20. Dependence of partial specific volume, υ, on the degree of polymerization n of PEtOx$_n$-lipids in solvent water. The dashed line indicates the partial specific volume of PEtOx.[3]

Figure S21 shows normalized distributions of intrinsic sedimentation coefficients, $c([s])$ (**eq. 3**) of PEtOx$_n$-lipids in ethanol (**Figure S21A**) and water (**Figure S21B**) as solvents from numerical analysis of sedimentation velocity analytical ultracentrifugation (SV-AUC) experiments. For all systems, a shift of the populations toward higher values of intrinsic sedimentation coefficients was observed when changing solvent from ethanol to water, indicating formation of aggregates in water.

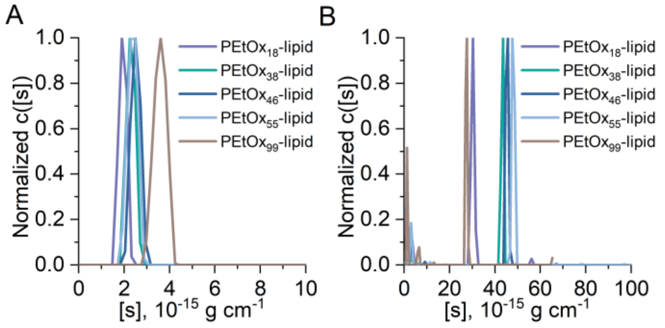

Figure S21. Differential distributions of intrinsic sedimentation coefficients, $c([s])$, of PEtOx$_n$-lipids in (**A**) ethanol and (**B**) water as solvents.

The plot of inverse sedimentation coefficients (eq. S3) against concentration for the PEtOx$_n$-lipids in solvent ethanol (**Figure S22A**) showed classical behavior for linear polymers. The sedimentation coefficients extrapolated to infinite dilution increased with an increase of polymer DP of the PEtOx$_n$-lipids (**Table S6**). In water, such patterns were observed as well (**Figure S22B**). The translational frictional ratios, f/f_{sph}, show an apparent absence of a concentration dependence in ethanol as the solvent (**Figure 23A**). For comparison, in water, f/f_{sph} values exhibit a stronger dependence, particularly with an increase of DP values (**Figure S23B, Table S6**).

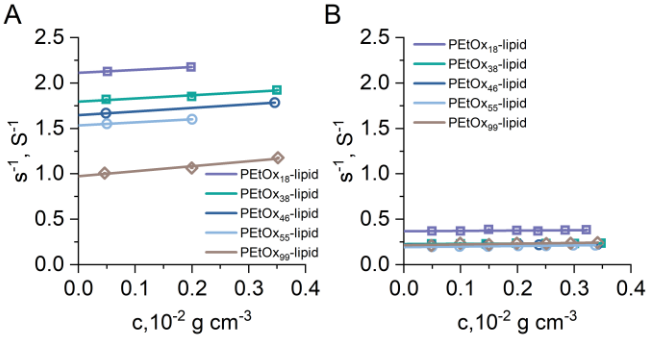

Figure S22. Concentration dependences of inverse sedimentation coefficients of PEtOx$_n$-lipids in (**A**) ethanol and (**B**) water as solvents.

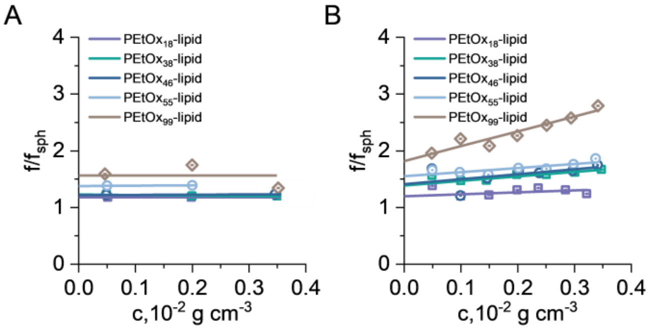

Figure S23. Concentration dependences of translational frictional ratios, f/f_{sph}, of PEtOx$_n$-lipids in (**A**) ethanol and (**B**) water as solvents.

The calculated molar masses based on sedimentation-diffusion analysis and the $c(s)$ model, $M_{s,f}$ (eq. S6), in ethanol as a solvent are listed in **Table S6**. Differences of determined molar masses indicate the formation of aggregates of the PEtOx$_n$-lipids in water with an aggregation number, N_{agg}, in the range from 19 to 127 (**Table S7**). The hydrodynamic characteristics of the PEtOx$_n$-lipid micelles assume values of A_0 in the typical range of polymers, with $A_0 = 3.39$ to 4.13 $g\ cm^2 s^{-2} K^{-1} mol^{-1/3}$ (**Table S6**).

Information about hydrodynamic sizes of the studied systems in water was obtained by combination of several orthogonal techniques. The hydrodynamic diameter, d_h, was calculated from sedimentation-diffusion analysis of SV-AUC experiments, $d_{h,AUC}$ (eq. S8) and obtained from intensity-based DLS ($d_{h,DLS}$) measurements. The resulting $c(d_h)$ and intensity distributions showed reasonable agreement with each other (**Figure S24**). Notably, the size distribution from DLS was wider compared with the diffusion-corrected distributions from SV-AUC experiments. Nevertheless, the resulting hydrodynamic diameter values correlated across both techniques (**Table S7**). Cryo-TEM measurements confirmed the formation of micelles in water (**Figure 4 E**). Values of hydration in water, δ, revealed a substantial difference in the values obtained through viscometric measurements (eq. 9) and through sedimentation-diffusion analysis (eq. 7, **Table S7**). However, a tendency of an increase of the average value of hydration with increasing DP was discernable from viscometric measurements (**Figure S25**), showing

that a spatial extension of the hydrophilic corona of the micelle allows accommodating larger amounts of water per micelle.

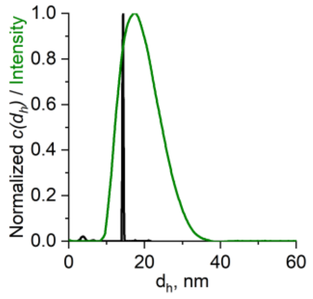

Figure S24. An example of size analysis of the $PEtOx_{46}$-lipid micelles in water. Normalized hydrodynamic size distributions, $c(d_h)$, from AUC (black curve) and intensity-based hydrodynamic size distributions from DLS (green curve).

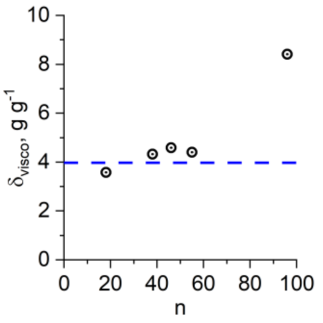

Figure S25. Dependence of hydration, δ_{visco}, on degree of polymerization (DP) indicated by n of the $PEtOx_n$-lipids in water as solvent. The dashed line indicates the average hydration value of commercial PEG-lipids.[2]

Table S6. Hydrodynamic characteristics of $PEtOx_n$-lipids in solvents ethanol and water.

$Sample$	[a] $[\eta]$, $cm^3 g^{-1}$	k_h	k_k	υ, $cm^3 g^{-1}$	s, S	f/f_{sph}	$M_{s.f}$, $g\ mol^{-1}$	$A_0, 10^{-10}$ $g\ cm^2 s^{-2} K^{-1} mol^{-1/3}$
Ethanol								
$PEtOx_{18}$-lipid	7.2	0.73	0.05	0.898	0.47	1.18	2,500	3.64
$PEtOx_{38}$-lipid	7.6	1.74	0.12	0.899	0.56	1.23	3,900	3.54
$PEtOx_{46}$-lipid	8.6	1.48	0.04	0.841	0.61	1.22	4,500	3.71
$PEtOx_{55}$-lipid	9.8	1.09	0.03	0.869	0.62	1.40	4,200	3.45
$PEtOx_{99}$-lipid	15.0	0.92	-0.07	0.888	1.03	1.72	14,700	3.19
Water								
$PEtOx_{18}$-lipid	11.3	-	-	0.915	2.70	1.20	167,000	4.13
$PEtOx_{38}$-lipid	13.1	-	-	0.904	4.41	1.46	389,000	3.59
$PEtOx_{46}$-lipid	13.8	-	-	0.902	4.57	1.41	377,000	3.78
$PEtOx_{55}$-lipid	13.3	-	-	0.904	5.14	1.55	535,000	3.39
$PEtOx_{99}$-lipid	23.2	-	-	0.848	4.63	1.81	284,000	3.58

[a]Average values obtained from Huggins and Kraemer.

Table S7. Hydrodynamic diameters and values of hydration of $PEtOx_n$-lipids in water.

$Sample$	[a] $d_{h,AUC}$ nm	$d_{h,AUC}$ nm	$d_{h,DLS}$ nm	N_{agg}	δ_{visco} g/g	δ_{AUC} g/g
$PEtOx_{18}$-lipid	2.3	9.4	7.0	66	3.58	0.66
$PEtOx_{38}$-lipid	2.8	15.1	10.9	100	4.33	1.91
$PEtOx_{46}$-lipid	2.9	14.5	13.6	85	4.59	1.62
$PEtOx_{55}$-lipid	3.2	17.9	13.1	127	4.41	2.46
$PEtOx_{99}$-lipid	6.0	16.5	15.7	19	8.41	4.17

[a]Values obtained in ethanol.

2.7.3. Hydrodynamic characterization of PEtOx46-lipids

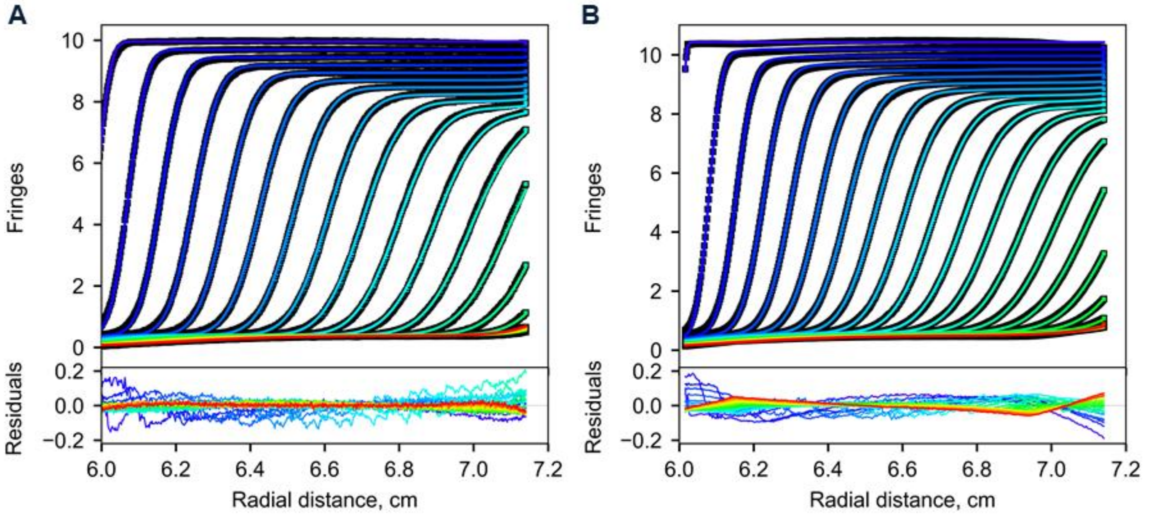

Figure S26. Radial distance scans at different times (black symbols) and numerical results from sedimentation−diffusion analysis, c(s), (colored lines) of (**A**) PEtOx46-lipid and (**B**) PEtOx46-lipid$_{clean}$ in water at a concentration of $c = 3.5\, mg\, ml^{-1}$.

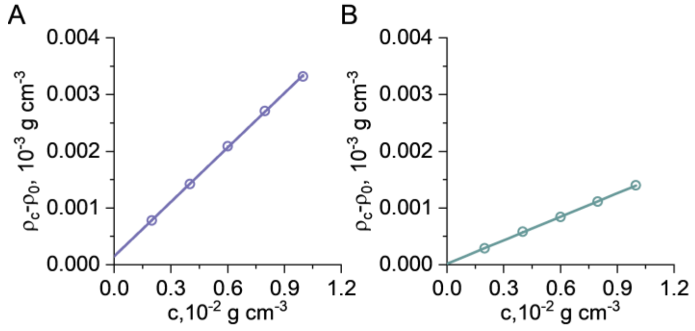

Figure S27. Density increment measurements, $\rho_c - \rho_0$, of PEtOx46-lipid$_{clean}$ in (**A**) ethanol and (**B**) water as solvents.

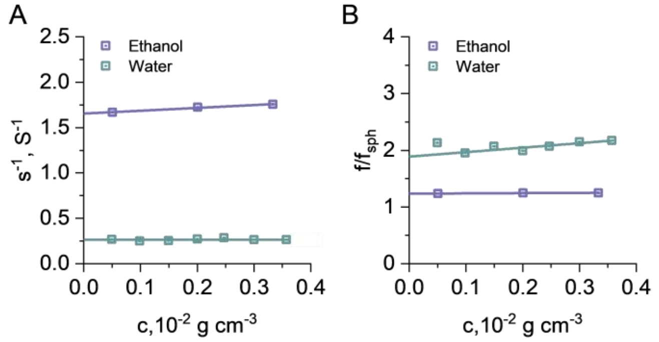

Figure S28. Concentration dependences of (**A**) inverse sedimentation coefficients, s^{-1}, and (**B**) translational frictional ratios, f/f_{sph}, of PEtOx46-lipid$_{clean}$ in ethanol and water as solvents.

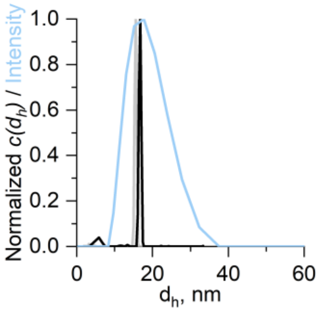

Figure S29. Normalized hydrodynamic size distributions in water obtained from sedimentation-diffusion analysis, $c(d_h)$, and intensity-based analysis from DLS (black and blue colors, respectively) of PEtOx$_{46}$-lipid$_{clean}$ in water. The size distribution in gray color originates from sedimentation-diffusion analysis, $c(d_h)$, obtained for PEG-lipid ALC-0159 in water from an independent previous study.[2]

Table S8. Hydrodynamic characteristics of previously studied commercial PEG-lipid systems (with a DP of 44)[2] and here studied $PEtOx_{46}$-lipids (DP 46) in ethanol and water as solvents.

$Sample$	$[\eta]$, $cm^3 g^{\cdot}$	k_h	k_k	υ, $cm^3 g^{\cdot}$	s, S	$[s], 10^{-15}$ $g\ cm^{-1}$	f/f_{sph}	$[D], 10^{-11}$ $cm\ g\ s^2\ K^{-1}$	$M_{s.f}$, $g\ mol^{-1}$	$A_0, 10^{-10}$ $g\ cm^2 s^{-2} K^{-1} mol^{-1/3}$
Ethanol										
ALC-0159[a]	6.7	0.69	0.05	0.90	0.38	1.7	1.18	6.99	2,000	3.55
DMG-PEG[a]	5.9	0.77	0.04	0.92	0.41	1.9	1.07	7.39	2,100	3.71
$PEtOx_{46}$-lipid	10.1	0.27	-0.20	0.92	0.61	2.7	1.22	5.11	4,500	3.71
$PEtOx_{46}$-$lipid_{clean}$	10.0	0.28	-0.19	0.86	0.6	2.4	1.23	5.63	3,500	3.95
Water										
ALC-0159[a]	12.2	-	-	0.89	2.62	24.8	1.85	0.86	224,000	2.75
DMG-PEG[a]	12.1	-	-	0.89	2.94	27.8	1.87	0.96	269,000	2.78
$PEtOx_{46}$-lipid	13.8	-	-	0.90	4.57	46.0	1.41	1.01	377,000	3.78
$PEtOx_{46}$-$lipid_{clean}$	12.5	-	-	0.86	3.77	26.8	1.89	0.88	254,000	2.77

[a] Values for ALC-0159 and DMG-PEG are published elsewhere.[2]

Table S9. Hydrodynamic diameters and values of hydration of previously studied commercial PEG-lipid systems (with a DP of 44)[2] and $PEtOx_{46}$-lipids (DP 46) in water.

$Sample$	[a]$d_{h,AUC}$ nm	$d_{h,AUC}$ nm	$d_{h,DLS}$ nm	$d_{cryo-TEM}$ nm	N_{agg}	δ_{visco} g/g	δ_{AUC} g/g
ALC-0159[b]	2.0	15.9	16.1	15.0	112	3.98	4.70
DMG-PEG[b]	2.0	17.1	16.5	15.1	128	3.96	4.90
$PEtOx_{46}$-lipid	2.9	14.5	13.6	12.5	85	4.59	1.62
$PEtOx_{46}$-$lipid_{clean}$	2.6	16.7	17.6	14.0	79	4.13	4.94

[a] Values obtained in ethanol

[b] Values for ALC-0159 and DMG-PEG are published elsewhere.[2]

3. References

(1) Holick, C. T.; Klein, T.; Mehnert, C.; Adermann, F.; Anufriev, I.; Streiber, M.; Harder, L.; Traeger, A.; Hoeppener, S.; Franke, C.; et al. Poly(2-ethyl-2-oxazoline) (POx) as Poly(ethylene glycol) (PEG)-Lipid Substitute for Lipid Nanoparticle Formulations. *Small* **2025**, *21* (16), 2411354. DOI: 10.1002/smll.202411354.

(2) Anufriev, I.; Hoeppener, S.; Nischang, I. PEG-Lipids: Quantitative Study of Unimers and Aggregates Thereof by the Methods of Molecular Hydrodynamics. *Anal. Chem.* **2023**, *95* (28), 10795-10802. DOI: 10.1021/acs.analchem.3c01999.

(3) Grube, M.; Leiske, M. N.; Schubert, U. S.; Nischang, I. POx as an Alternative to PEG? A Hydrodynamic and Light Scattering Study. *Macromolecules* **2018**, *51* (5), 1905-1916. DOI: 10.1021/acs.macromol.7b02665.
